# Identification of Nasal Gammaproteobacteria with Potent Activity against Staphylococcus aureus: Novel Insights into the “Noncarrier” State

**DOI:** 10.1128/mSphere.01015-20

**Published:** 2021-01-06

**Authors:** Amy L. Cole, Meera Sundar, Ana Lopez, Anna Forsman, Shibu Yooseph, Alexander M. Cole

**Affiliations:** a Burnett School of Biomedical Sciences, College of Medicine, University of Central Florida, Orlando, Florida, USA; b Department of Computer Science, College of Engineering and Computer Science, University of Central Florida, Orlando, Florida, USA; c Genomics and Bioinformatics Faculty Cluster, University of Central Florida, Orlando, Florida, USA; d Department of Biology, College of Sciences, University of Central Florida, Orlando, Florida, USA; University of Kentucky

**Keywords:** *Staphylococcus aureus*, nasal carriage, microbiome, bacterial interference

## Abstract

Staphylococcus aureus nasal carriage provides the bacterial reservoir for opportunistic infection. In comparing the nasal microbiomes of culture-defined persistent S. aureus carriers versus noncarriers, we detected S. aureus DNA in all noses, including those with an established history of S. aureus negativity based on culture. Colonization with *Gammaproteobacteria*, including Klebsiella aerogenes, Citrobacter koseri, Moraxella lincolnii, and select Acinetobacter spp., was associated with S. aureus noncarriage. We next developed physiological competition assays for testing anti-S. aureus activity of isolated nasal species, utilizing medium modeling the nutrient-limited fluid of the nasal mucosa, polarized primary nasal epithelia, and nasal secretions. K. aerogenes from the nose of an S. aureus noncarrier demonstrated >99% inhibition of S. aureus recovery in all assays, even when S. aureus was coincubated in 9-fold excess. Secreted S. aureus inhibitory proteins from K. aerogenes and *M. lincolnii* were heat-stable and <30 kDa, fitting the profile of antimicrobial peptides. *C. koseri*, Acinetobacter haemolyticus, Acinetobacter junii, and Acinetobacter schindleri inhibited S. aureus recovery on nasal epithelia in a contact-dependent manner, while several other species either had no effect or promoted S. aureus growth. Collectively, this project is one of the first to identify resident nasal microbial species that impede S. aureus survival, and it implies that detectable nasal S. aureus results from shifts in microbial community composition.

**IMPORTANCE** Nasal carriage of Staphylococcus aureus is a risk factor for infection, but it is not yet understood why some individuals carry nasal S. aureus persistently, intermittently, or seemingly not at all when tested via culture methods. This study compared the nasal microbiomes of established S. aureus carriers and noncarriers, identified species associated with noncarriage, and tested them for anti-S. aureus activity using assays developed to model the nutrient-limited nasal mucosa. We determined that all nostril swabs contain S. aureus DNA, even swabs from hosts considered to be long-term noncarriers. Select members of the *Gammaproteobacteria* class were more prevalent in noncarrier than carrier nostrils and demonstrated potent activity against multiple strains of S. aureus. The results described here provide a better understanding of how the nasal microbiome controls S. aureus growth and viability and may be useful in the design of improved S. aureus decolonization strategies.

## Abstract (precis)

Nasal carriage of Staphylococcus aureus is a risk factor for infection, but it is not yet understood why some individuals carry nasal S. aureus persistently, intermittently, or seemingly not at all when tested via culture methods. This study compared the nasal microbiomes of established S. aureus carriers and noncarriers, identified species associated with noncarriage, and tested them for anti-S. aureus activity using assays developed to model the nutrient-limited nasal mucosa.

## INTRODUCTION

Staphylococcus aureus infections have been a concern for health care for decades, especially when considering the rise in antibiotic-resistant strains and postoperative S. aureus infections that plague hospital systems worldwide (1, 2). While S. aureus is not generally considered to be a main colonizer of the human gut (3), it resides at least transiently in the moist mucosa of the anterior nares (3–6). S. aureus nasal carriage (SANC) is typically asymptomatic, but transmission of S. aureus from the nasal cavity to other areas of the body renders carriers more prone to developing S. aureus infections (7, 8). Nasally carried S. aureus is also frequently spread to household members and domestic pets (9–11). On account of this and the finding that even newborns are S. aureus colonized (12), S. aureus should be considered ubiquitous, and investigations of carriage dynamics warrant high priority.

Previous studies have suggested that SANC is determined by host and environmental factors rather than by individual strain characteristics of the bacterium (13–15). Higher carriage rates are found in infants, toddlers, and the elderly; risk factors for adults include HIV, type II diabetes, atopic dermatitis, autoimmune disease, rheumatoid arthritis, cancer, prolonged hospital stays, and close living quarters such as prisons, nursing homes, and daycare centers (16–20). Smoking has been linked with elevated nasal S. aureus load and a decreased capacity for clearing nasal S. aureus (21). Conventionally, SANC is categorized according to frequency of S. aureus detection by culture methods during repeat sampling over months and years. Nostril swabs of persistent carriers always contain detectable S. aureus, and intermittent carriers periodically clear S. aureus to undetectable levels, while noncarriers lack detectable S. aureus (22, 23). We demonstrated that nasal fluids of human S. aureus carriers have less intrinsic anti-S. aureus activity than nasal fluids collected from noncarriers (15, 24, 25) even though the basal components of nasal secretions (e.g., amino acids, salts, organic acids, trace elements, sugars) are not variable between donors (26). Host antimicrobial peptide polymorphisms fail to distinguish S. aureus carriers from noncarriers, and HNP1 to -3 and various cytokine levels are elevated in carriers, suggesting that these proteins are more the result of SANC than a reason for noncarrier status (13, 15, 21, 27). Collectively, these insights led us to the current question of how the constantly fluctuating nasal microbiota affect SANC status.

The human nasal microbiome consists mainly of *Corynebacterium* (phylum *Actinobacteria*), *Staphylococcus* (phylum *Firmicutes*), and *Cutibacterium* (formerly *Propionibacterium*; phylum *Actinobacteria*), balanced by lower levels (typically) of various *Proteobacteria* as well as *Dolosigranulum* and *Streptococcus* genera of the phylum *Firmicutes*. Nasal microbiome dynamics appear to be less determined by host genetics than by environment, with fluctuations influenced by hygiene, climate, diet, prescription drugs, and interpersonal relationships (28, 29). At the species level, the role of the nasal microbiome in promoting or preventing SANC remains mostly unknown. Conflicting reports have emerged regarding the role of S. epidermidis (28, 30, 31). S. lugdunensis, S. hominis, and S. saprophyticus have demonstrated promise as anti-S. aureus effectors (31, 32), but it remains unclear if they are present in high enough abundance to distinguish SANC from non-SANC individuals. Corynebacterium pseudodiphtheriticum produces a factor(s) with bactericidal activity toward S. aureus in *in vitro* agar-based zone of clearance assays (33). However, the relative abundance of this species was found to be inversely related to S. aureus in ciliated respiratory epithelia but not in the anterior nares (34). Here, we evaluated the nasal microbiomes of the three SANC subgroups using 16S rRNA amplicon sequencing and also compared noncarrier versus persistent nostrils at the species level using culture methods and shotgun DNA metagenomic sequencing. Notably, certain *Proteobacteria* were more strongly associated with noncarriers. We developed physiologically relevant assays for testing nasal S. epidermidis, C. pseudodiphtheriticum, and several nasal strains of *Klebsiella*, *Citrobacter*, Acinetobacter, and *Moraxella* spp. for inhibitory activity against nasal S. aureus.

## RESULTS

### Nasal S. aureus load correlates with *in vitro* growth of S. aureus in nasal secretions.

**FIG 1:**
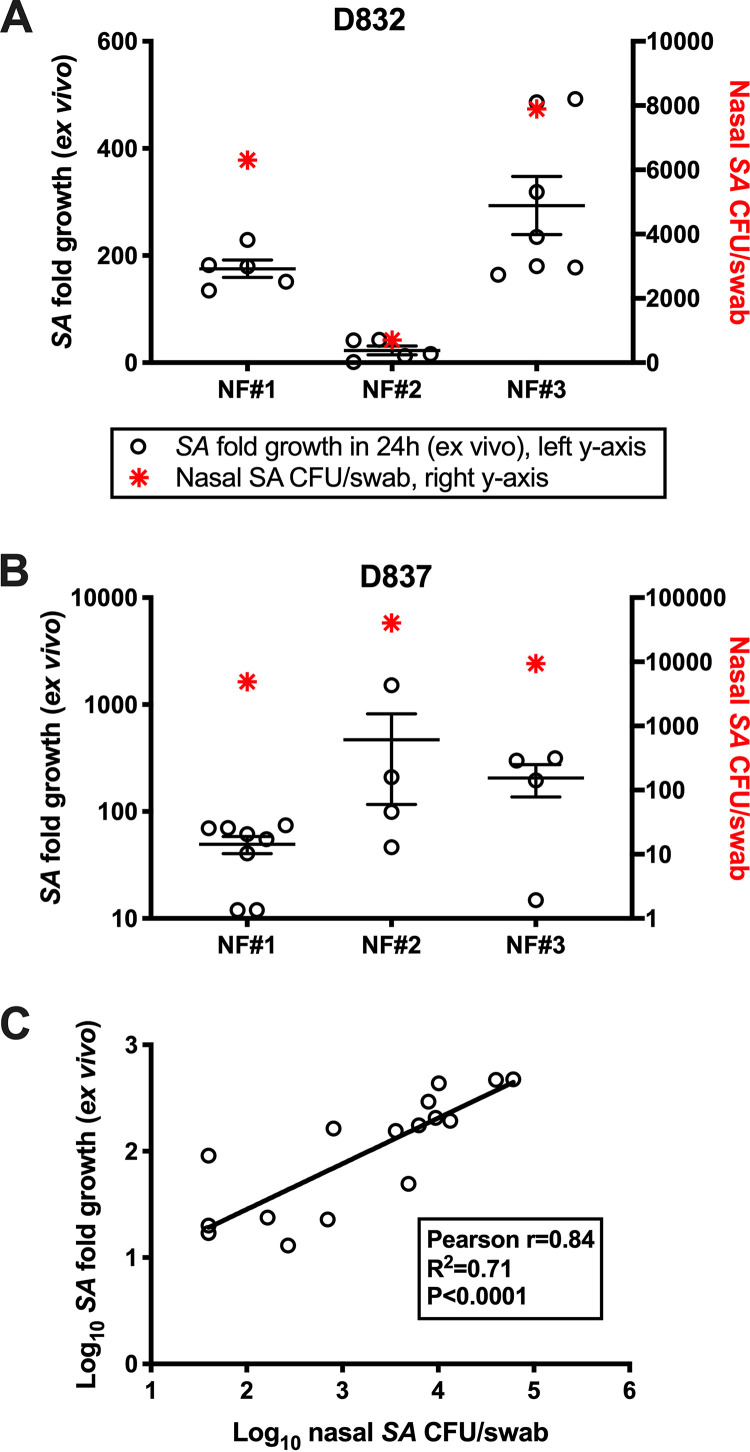
Growth of S. aureus in nasal secretions correlates with host nasal S. aureus load. Subjects self-collected nasal swabs and secretions every 4 to 7 days. (A and B) Donors 832 (A) and 837 (B) were persistent S. aureus carriers (all swabs positive for S. aureus as determined by CFU) for over a year. Open circles represent the fold growth (left *y* axis) of unique nasal S. aureus strains in the donor’s nasal fluids that were collected in different weeks (NF no. 1 to 3, *x* axis). Red asterisks represent the donor nasal S. aureus load (right *y* axis) matching the day the NF was collected. (C) Nasal S. aureus load (*x* axis, log scale) versus S. aureus fold growth in NF *ex vivo* (*y* axis, log scale) for 17 unique nasal fluids. Correlation coefficient (Pearson’s r) and statistics are shown in the box, as is a regression line depicting the positive correlation.

We demonstrated previously that nasal secretions from S. aureus carriers support the *ex vivo* growth of S. aureus more than secretions form healthy noncarriers (4, 15, 24). However, host antimicrobial peptides, including HNP-1, HNP-3, and HBD-2, are elevated in S. aureus carrier fluids compared to noncarrier fluids (4, 13). This indicates that host expression of known anti-S. aureus effectors is more likely the result of S. aureus colonization than a reason that some individuals appear not to carry S. aureus. To further evaluate the role of secreted factors in modulating nasal S. aureus load, we measured the growth of five different S. aureus isolates in S. aureus carrier secretions collected at various time points when the corresponding nasal S. aureus load was known. Figure 1A and B demonstrate the association between nasal S. aureus load and growth of S. aureus in matched nasal secretions for two donors (D832 in Fig. 1A and D837 in Fig. 1B). These subjects were in good health and were categorized as persistent S. aureus carriers by presenting S. aureus-positive nasal swabs weekly for over a year. In 17 nasal fluid samples collected from nasal carriers in which a matched nasal S. aureus count was available, the average growth of 5 S. aureus strains correlated with the nasal load of the host nose (Fig. 1C; Pearson *r* = 0.84, *P < *0.0001). Collectively, this suggested that nasal secretions contain factors that dictate how much S. aureus is detectable by culture methods. Considering that nasal secretions have been demonstrated to be indistinguishable between S. aureus carriers and noncarriers in terms of nutrient and metabolite composition (26), it became evident that nasal microbiome composition might control nasal S. aureus survival.

### Genus-level nasal microbial community profiling reveals no appreciable difference between nasal S. aureus carriers and noncarriers.

**FIG 2:**
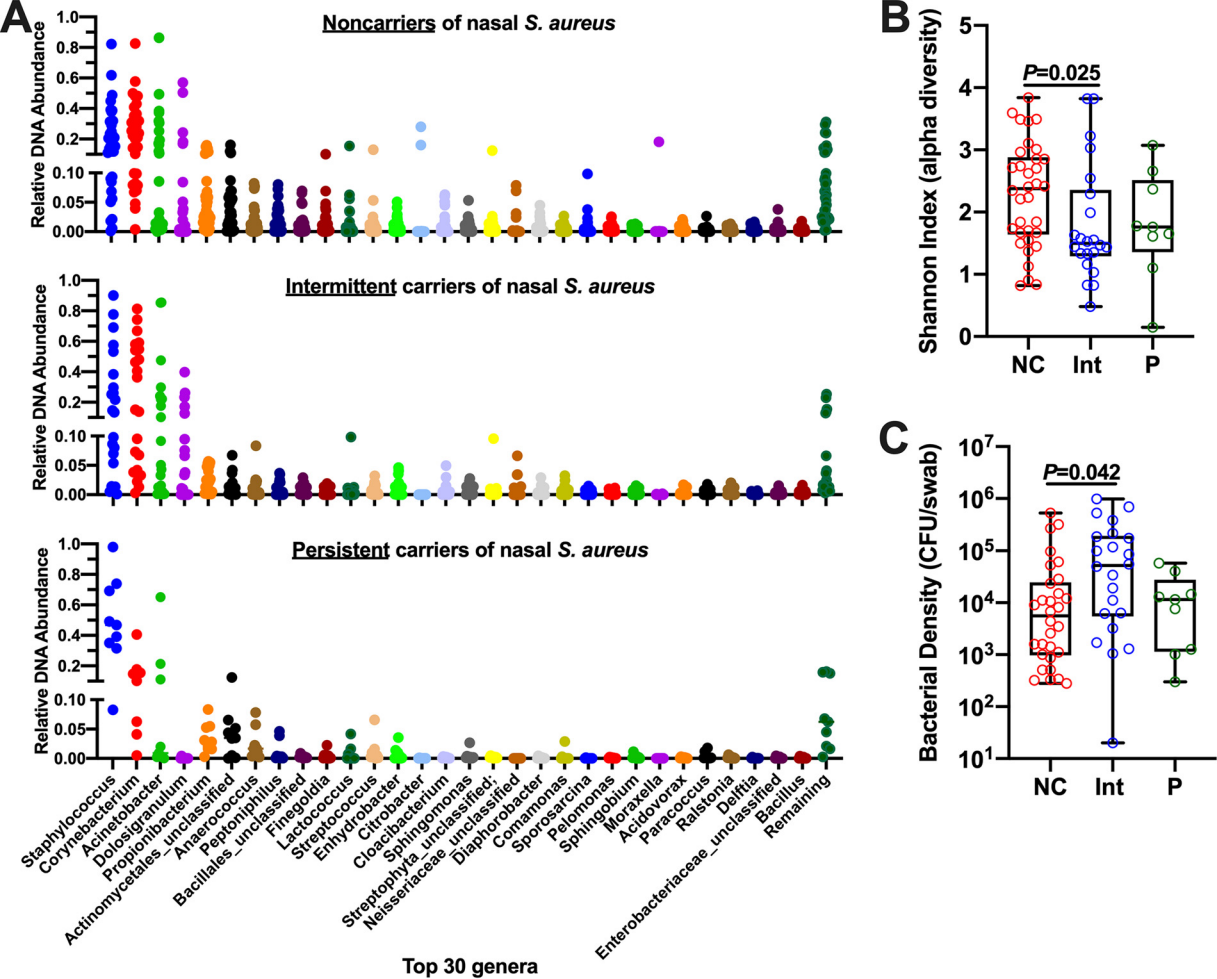
Genus-level microbial composition of non-SANC, persistent SANC, and intermittent SANC nostrils. (A) Relative abundance of genera detected by 16S amplicon sequencing of nostril swabs collected from 34 noncarrier nostrils, 22 intermittent (11 S. aureus-positive/11 S. aureus-negative) SANC nostrils, and 9 persistent SANC nostrils. (B) Shannon Index (alpha diversity) for noncarriers (NC), intermittent carriers (Int), and persistent carriers (P), calculated from the OTU data for each sample. (C) Bacterial density of each swab, grouped according to SANC status as in panel B. Panels B and C show individual values as well as box and whisker representation of the median, 25th and 75th percentiles (boxes), and minimum/maximum values (whiskers). *P* values were calculated using the Wilcoxin rank-sum test (panel B) and ordinary one-way ANOVA (panel C).

We next sought to identify the abundant species present in the noses of individuals considered to be S. aureus noncarriers as detected by culture methods and to characterize which species levels fluctuate when intermittent carriers cleared or nearly cleared S. aureus. Nasal swabs were vortexed in broth and spread onto 6 different agars, including both nonselective (plate count, nutrient, Trypticase soy agar [TSA], TSA II/5% blood) and S. aureus-selective (mannitol salt agar or CHROMagar S. aureus) formulations. Even in demonstrated S. aureus noncarriers, it quickly became apparent that non-*Staphylococcus* genera account for a minority of visible colonies when nutrient-rich agars are employed. Out of 60 colonies (identified by full-length 16S sequencing), only 5 non-*Staphylococcus* spp. were identified (Micrococcus luteus, Micrococcus yunnanensis, Pantoea septica, Klebsiella aerogenes, and Enterococcus faecalis), while S. aureus (in carrier noses), S. epidermidis, and S. hominis accounted for most colonies identified. In an effort to detect more *Proteobacteria* and *Actinobacteria* species in noses with known SANC status, we next performed microbial community profiling of nasal swab DNA. 16S rRNA amplicon sequencing (Illumina MiSeq) was performed on 65 nasal swab DNA extracts collected from 25 individuals over time—34 swabs from noncarrier nostrils (15 individuals), 9 swabs from persistent S. aureus carrier nostrils (4 individuals), and 22 (11 S. aureus-negative/11 S. aureus-positive) swabs from intermittent carrier nostrils (6 individuals). As shown in Fig. 2A, taxonomic profiling at the genus level revealed that all noses are heavily colonized with *Staphylococcus* and *Corynebacterium* regardless of SANC status and that a majority of noses also contain Acinetobacter, *Dolosigranulum*, and a mixture of low-abundance genera from the *Actinobacteria*, *Firmicutes*, and *Proteobacteria* phyla. A stacked bar representation of each nostril’s taxonomic profile (*n* = 65) is provided as Fig. S1. Shannon diversity analysis revealed that noncarriers exhibited more diverse taxa than the other groups, although only the difference between noncarriers and intermittent carriers reached statistical significance (Fig. 2B; and P = 0.025). Total bacterial density (CFU/swab) ranged widely for all groups (10^2^ to 10^6^ CFU/swab) and trended higher for intermittent versus noncarriers (Fig. 2C; and P = 0.042), suggesting that noncarriers’ increased microbial diversity was not due simply to enhanced bacterial load. There was no difference in the number of sequence reads between groups (not shown). The 65 samples were clustered based on their genus-level taxonomic profiles using the Dirichlet multinomial mixture (DMM) model framework (35), and this analysis revealed the presence of two clusters, a *Staphylococcus*-prevalent cluster and a *Corynebacterium*-prevalent cluster. Each of these contained a mixture of noncarriers, intermittent carriers, and persistent carriers (not shown). Stratifying the intermittent carrier nostril taxa according to SANC status (11 S. aureus-negative versus 11 S. aureus-positive swabs) also revealed no appreciable differences between the nasal microbial communities (Fig. S2). Taken together, these data corroborate the findings of Piewngam et al. (36), who also reported that genus-level profiling does not provide the resolution required to distinguish SANC from non-SANC individuals.

10.1128/mSphere.01015-20.1 FIG S1 Genus-level microbial composition of non-SANC, persistent SANC, and intermittent SANC nostrils. Relative abundance of genera detected by 16S amplicon sequencing of nostril swabs collected from 34 noncarrier (NonSANC) nostrils, 9 persistent SANC nostrils, and 22 intermittent (11 S. aureus-positive/11 S. aureus-negative) SANC nostrils. The horizontal lines beneath the bars indicate swabs collected from the same donor over time. The top 30 detected genera and color representation are indicated at right. Download FIG S1, PDF file, 0.1 MB. Copyright © 2021 Cole et al. 2021 Cole et al. This content is distributed under the terms of the Creative Commons Attribution 4.0 International license.

10.1128/mSphere.01015-20.2 FIG S2 Genus-level microbial composition of intermittent SANC nostrils. Relative abundance of genera detected by 16S amplicon sequencing of nostril swabs collected from intermittent S. aureus carriers. (Top) 11 S. aureus-negative intermittent nostrils. (Bottom) 11 S. aureus-positive intermittent nostrils. The top 30 genera detected in human nostrils are shown, along with remaining reads shown in dark green. Download FIG S2, PDF file, 0.1 MB. Copyright © 2021 Cole et al. 2021 Cole et al. This content is distributed under the terms of the Creative Commons Attribution 4.0 International license.

### Identification of species associated with S. aureus culture-negative noses.

We next took a two-pronged approach to identifying nasal microbiome species that might disfavor S. aureus colonization. Six subjects, each of whom had submitted regular swabs for at least 3 months and thus presented a known SANC status, self-collected nasal swabs twice weekly. With the first swab, SANC status was confirmed by identification of S. aureus colonies on CHROMagar S. aureus plates, and in parallel, isolation of non-*Staphylococcus* species was attempted by culturing nasal samples on MacConkey II and Leeds agars, which favor growth of Gram-negative species. The six subjects comprised 3 noncarriers, 2 persistent carriers, and 1 carrier with an ∼80% S. aureus-positive swab rate over 3 years of monitoring. In general, noncarrier donor swabs yielded tens to hundreds of colonies on MacConkey II and Leeds agars, while SANC donor swabs yielded no CFU on MacConkey II agar and few CFU on Leeds agar. Dozens of colonies were plucked and cultured overnight in nutrient broth, followed by identification by 16S PCR/Sanger sequencing. The most common isolate from noncarrier noses was Klebsiella aerogenes, followed by Citrobacter koseri and Klebsiella oxytoca. For the Leeds agar CFU cultured from S. aureus-positive swabs, nearly all colonies (18 of 21) were identified as *Staphylococcus* (*epidermidis*, *haemolyticus*, and *hominis*), and no *Klebsiella* or *Citrobacter* species were detected.

**FIG 3:**
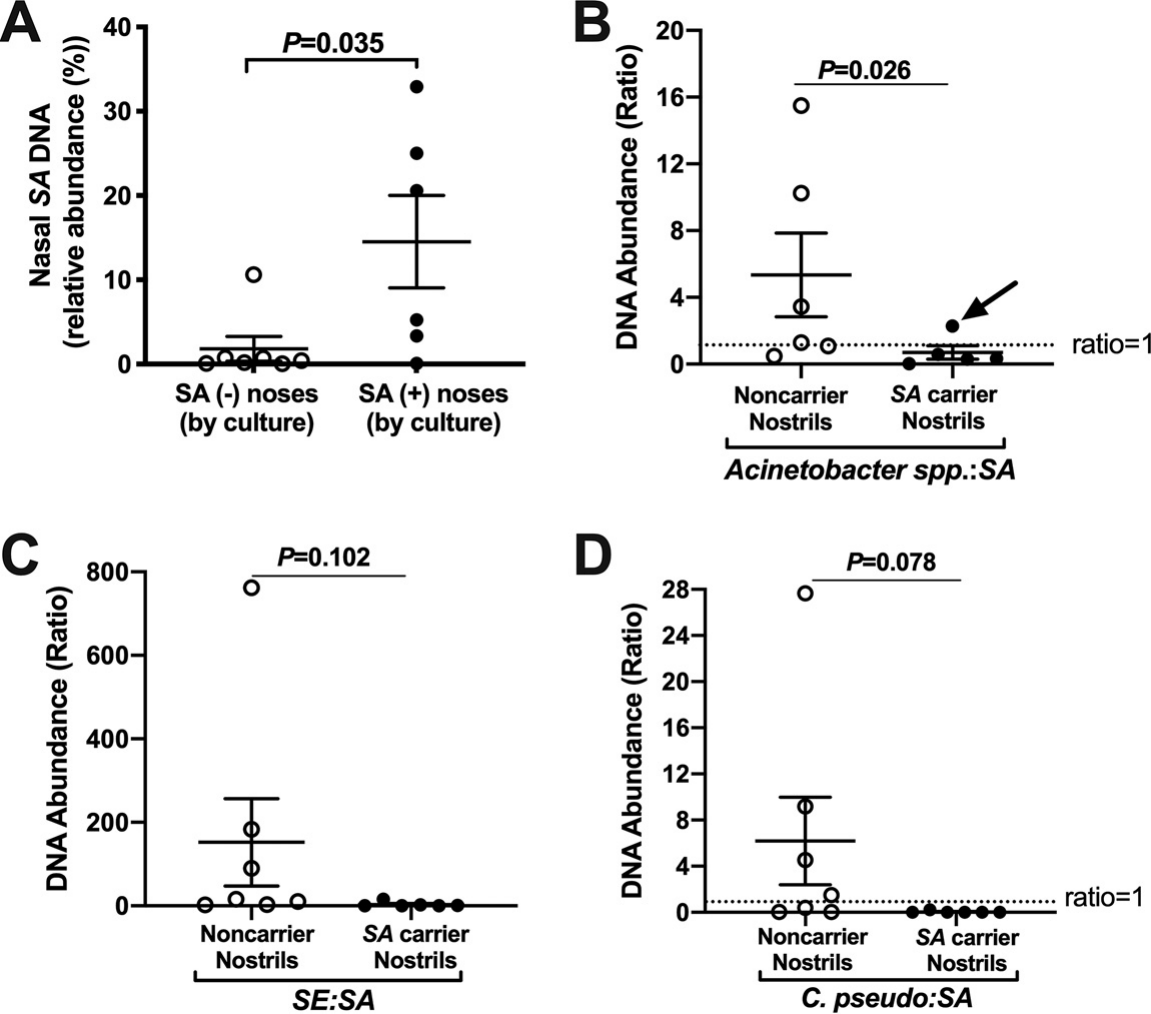
Species-level nasal microbial community analysis reveals S. aureus DNA in all nostrils. Subjects with known SANC status, as determined by culture, self-collected fresh nasal swabs which were immediately processed for total DNA extraction followed by microbial DNA enrichment. Shotgun sequencing was performed for 7 “noncarrier” and 6 S. aureus carrier nostrils. (A) Reads mapping to S. aureus (SA) genomes (strains) were present in all nostrils regardless of culture-defined SANC status. (B) Acinetobacter spp.: S. aureus ratio for noncarrier versus S. aureus carrier nostrils. The arrow indicates a carrier who nearly cleared S. aureus 4 days later. (C and D) DNA abundance ratio of S. epidermidis (*SE*, panel C) and C. pseudodiphtheriticum (D) to S. aureus for the same groups. Error bars represent the mean ± the standard error of the mean (SEM). *P* values are indicated for each comparison (unpaired *t* tests).

Subsequent nasal swab collections, occurring 2 to 3 days after cultured swabs, were immediately processed for DNA isolation coupled with a mammalian DNA depletion step. The resulting DNA was fragmented and indexed, and 14 samples were pooled and sequenced (single-lane HiSeq 2 × 150 bp). The 14 samples were from 7 noncarrier and 6 SANC nostrils, in addition to a microbial community standard that served to validate the library preparation and sequencing resolution. In total, 416 unique species were detected, while about 90 species were common to all noses. A list of the most prevalent genera and species, and the relative abundance and number of colonized nostrils in each group, is provided (Table 1). All seven culture-defined non-SANC nostrils contained S. aureus DNA (average relative abundance, 1.83%), while SANC nostrils averaged 14.53% S. aureus DNA (Fig. 3A and Table 1). Several *Staphylococcus* and *Corynebacterium* species appeared to associate with noncarrier noses, including *S. capitis*, S. epidermidis, *S. lugdunensis*, Corynebacterium accolens, Corynebacterium segmentosum, and C. pseudodiphtheriticum (Table 1). However, due to the variation between nostrils from different hosts, differences in abundance of these species between persistent and noncarriers did not reach statistical significance. Instead, we evaluated the ratio of various species to S. aureus for individual nostrils in each group. For Acinetobacter spp., whose relative DNA abundances were low and lacked consensus (only A. junii was present in all seven noncarrier nostrils, while no Acinetobacter spp. were common to all SANC nostrils [Table 1]), the ratio of total Acinetobacter to S. aureus was calculated. Six of seven noncarrier noses exhibited an Acinetobacter:S. aureus ratio of >1, while 5 of 6 carrier ratios were <1 (Fig. 3B; and P = 0.026). The carrier nose in which Acinetobacter outnumbered S. aureus (arrow in Fig. 3B) nearly cleared S. aureus according to a swab sample collected 4 days later. The S. epidermidis:S. aureus and Corynebacterium pseudodiphtheriticum:S. aureus ratios trended higher for noncarrier noses but did not reach statistical significance (Fig. 3C and D), nor did any other species:S. aureus DNA abundance ratio. Members of the *Gammaproteobacteria* class, including Citrobacter koseri, Klebsiella aerogenes, and Moraxella lincolnii, resided more often in non-SANC nostrils (Table 1). These species along with S. epidermidis, C. pseudodiphtheriticum, and a variety of Acinetobacter spp. were next tested for anti-S. aureus activity.

**TABLE 1:** Nasal microbial composition of culture-defined noncarriers and carriers of S. aureus^*a*^

Genus and species	Noncarrier noses		S. aureus carrier noses	
	Avg. relative abundance (%)	No. of colonized nostrils	Avg. relative abundance (%)	No. of colonized nostrils
Staphylococcus				
S. aureus	1.829	7 of 7	14.528	6 of 6
S. auricularis	0.024	3 of 7	0.614	3 of 6
S. capitis	1.936	7 of 7	0.439	5 of 6
S. cohnii	0.064	3 of 7	0.006	1 of 6
S. epidermidis	16.669	7 of 7	9.140	6 of 6
S. equorum	0.014	2 of 7	Und	0 of 6
S. haemolyticus	0.050	5 of 7	0.461	5 of 6
S. hominis	0.088	6 of 7	0.085	6 of 6
S. lugdunensis	1.263	5 of 7	0.493	4 of 6
S. pasteuri	0.410	7 of 7	0.338	5 of 6
S. saprophyticus	0.006	1 of 7	0.009	1 of 6
S. warneri	0.363	5 of 7	0.606	5 of 6
*Corynebacterium*				
C. accolens	13.927	7 of 7	3.367	6 of 6
C. segmentosum	13.737	7 of 7	3.190	6 of 6
C. aurimucosum	6.783	7 of 7	12.992	6 of 6
C. pseudodiphtheriticum	1.617	7 of 7	0.064	3 of 6
C. matruchotii	1.024	2 of 7	0.206	3 of 6
C. stationis	0.134	3 of 7	0.006	1 of 6
C. diphtheriae	0.199	6 of 7	0.058	6 of 6
C. kroppenstedtii	0.509	6 of 7	0.706	5 of 6
C. simulans	0.109	6 of 7	0.033	5 of 6
C. ureicelerivorans	0.036	5 of 7	0.017	2 of 6
C. otitidis	0.023	3 of 7	0.007	1 of 6
C. fournierii	0.020	3 of 7	0.006	1 of 6
C. resistens	0.015	4 of 7	Und	0 of 6
Cutibacterium acnes	9.339	7 of 7	8.590	6 of 6
Cutibacterium granulosum	1.113	7 of 7	3.233	6 of 6
Dolosigranulum pigrum	2.046	7 of 7	0.273	1 of 6
Citrobacter koseri	0.464	2 of 7	Und	0 of 6
Klebsiella aerogenes	0.174	3 of 7	0.006	1 of 6
Moraxella lincolnii	9.296	6 of 7	0.071	1 of 6
Bacillus subtilis	0.070	3 of 7	1.025	4 of 6
*Streptococcus* spp.	0.370	5 of 7	3.172	6 of 6
Acinetobacter				
A. baumannii	0.455	6 of 7	1.453	3 of 6
A. haemolyticus	0.008	2 of 7	0.013	3 of 6
A. johnsonii	0.097	5 of 7	0.208	3 of 6
A. junii	0.750	7 of 7	1.501	5 of 6
A. lwoffii	0.110	5 of 7	0.069	5 of 6
A. pittii	0.148	5 of 7	0.074	3 of 6
A. radioresistens	0.006	2 of 7	0.020	2 of 6
A. schindleri	0.094	5 of 7	0.102	2 of 6
A. towneri	0.013	3 of 7	0.037	2 of 6
A. venetianus	0.087	3 of 7	0.141	1 of 6
A. wuhouensis	0.114	2 of 7	0.028	2 of 6

a Und, undetected in all swabs within the indicated group.

### Survival of S. aureus in synthetic nasal medium is potently inhibited by K. aerogenes, *C. koseri*, and *M. lincolnii*.

**FIG 4:**
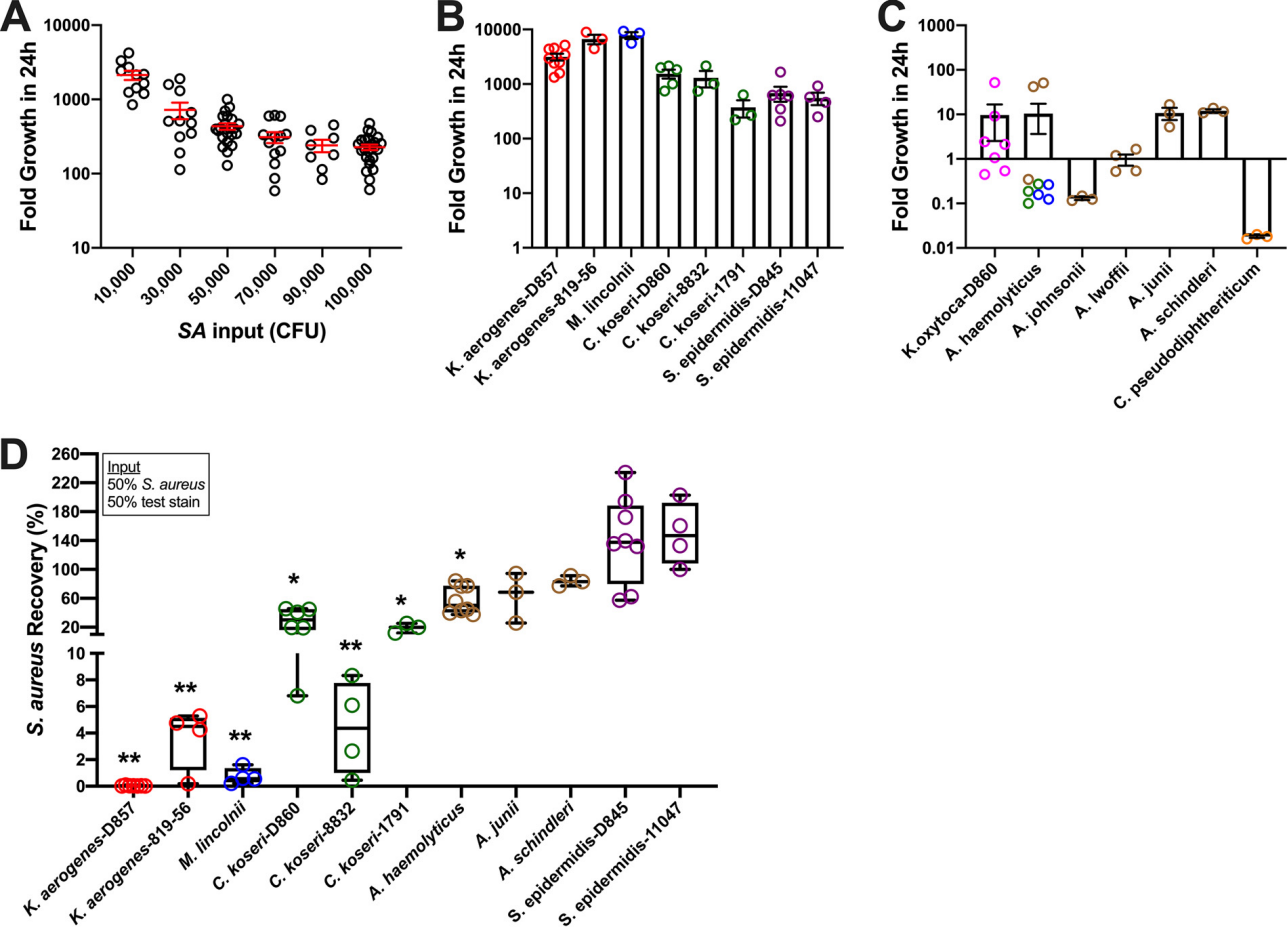
Inhibited S. aureus survival in nasal medium containing select species from the *Gammaproteobacteria* class. (A) Multiple unique strains of S. aureus were tested for their ability to proliferate in a physiological synthetic nasal medium supplemented with 0.1% human serum (SNM). Error bars represent the mean ± SEM. (B and C) Growth of putative nasal S. aureus competitor strains in SNM. Individual data points are shown as open circles. Bars and error bars indicate the mean ± SEM. Colored circles for *A. haemolyticus* indicate the unique strains tested. (D) To screen which resident nasal species compete with S. aureus, mixtures (50,000 S. aureus CFU + 50,000 CFU test strain) were incubated, and S. aureus recovery was calculated by comparing S. aureus CFU on selective agar when S. aureus was incubated alone versus with the indicated strain. Individual data points and box and whiskers representing the median, 25th to 75th percentiles, and min/max values are shown for at least 3 experiments using unique nasal strains of S. aureus. *, *P* < 0.05; **, *P* < 0.01 versus S. aureus incubated alone (100% recovery).

We screened 11 nasal bacterial species (17 strains) for inhibitory activity against S. aureus using a nutrient background composed of synthetic nasal medium (26) supplemented with 0.1% human serum (here referred to as SNM). Krismer et al. found that S. aureus growth in SNM led to gene expression patterns similar to what was observed during *in vivo* colonization (26). All strains (S. aureus and putative competitors) were isolated from nostril swabs performed in our laboratory or were nasal isolates purchased from the American Type Culture Collection (ATCC). To reduce growth variations due to differences in isolation procedures or broths used for initial stock preparations and subcultures, all strains were propagated in SNM on a layer of primary nasal epithelium, washed and separated from nasal cells, and stored frozen in single-use aliquots. Five genetically distinct nasal S. aureus strains from our carrier cohort demonstrated the capacity to proliferate 1,000- to 10,000-fold in SNM in 24 h, while growth rates plateaued at input concentrations greater than 10^5^ CFU/ml (50,000 CFU/0.5 ml in Fig. 4A). The growth rates of K. aerogenes, *C. koseri*, *M. lincolnii*, and S. epidermidis also exceeded 100-fold using an input of 10^5^ CFU/ml (Fig. 4B), while Acinetobacter strains, C. pseudodiphtheriticum, and K. oxytoca did not thrive in SNM (Fig. 4C). K. aerogenes collected from donor 857, an established noncarrier in our cohort, inhibited the recovery of all 5 S. aureus strains by over 99% when incubated as a 50:50 mixture (input = 50,000 CFU/well each bacterium; Fig. 4D). K. aerogenes-819-56 inhibited S. aureus >95%, as did *M. lincolnii*. S. aureus recovery was <40% in cocultures with *C. koseri* isolated from donor 860 or with *C. koseri*-1791 (ATCC); and S. aureus recovery was <10% with *C. koseri*-8832 (ATCC). For nasal strains of Acinetobacter, *A. haemolyticus* inhibited S. aureus recovery by ∼40% (Fig. 4D), even though it did not propagate as well as S. aureus in SNM (Fig. 4C). Notably, neither strain of nasal S. epidermidis (D845 isolated in our lab or 11047 purchased from ATCC) attenuated S. aureus recovery (Fig. 4D).

**FIG 5:**
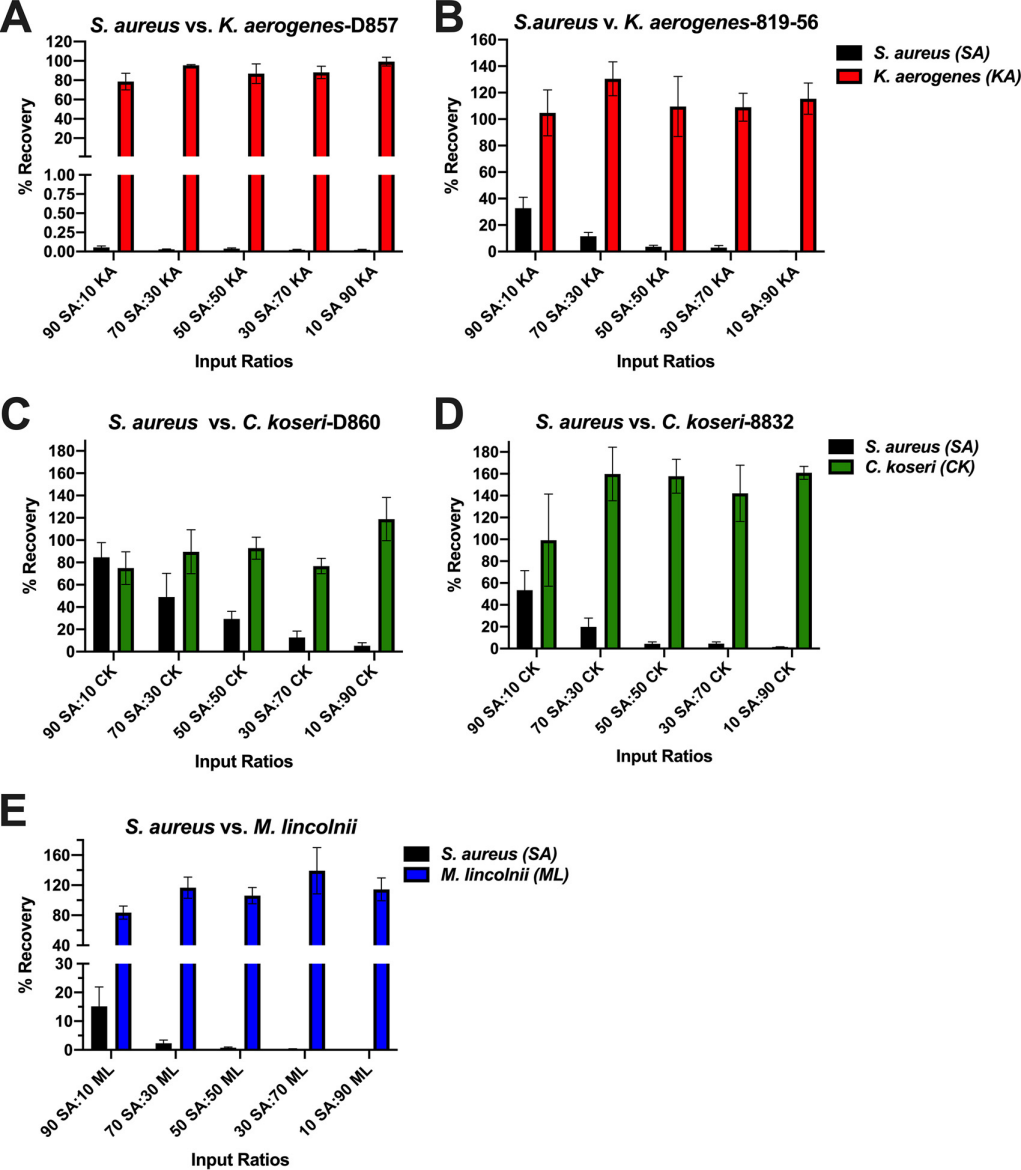
Potent inhibition of nasal S. aureus by K. aerogenes, *C. koseri*, and *M. lincolnii*. S. aureus and the indicated strains were incubated in SNM using the presented input ratios spanning from 90% S. aureus:10% competitor to 10% S. aureus:90% competitor, and CFU on selective agars were enumerated after 24 h. Recovery (%) was calculated by comparing the CFU yield from the coincubations versus incubations of 100% S. aureus or 100% test strain. Bars represent the mean ± SEM for at least 5 independent experiments using unique strains of nasal S. aureus.

Competitor strains, determined to have potent anti-S. aureus activity in the 50:50 assay, were tested next at input ratios spanning from 90% S. aureus:10% competitor to the inverse (10% S. aureus:90% competitor; Fig. 5). Compared to incubation of S. aureus alone, a mixture containing 10% K. aerogenes-D857 and 90% S. aureus reduced S. aureus recovery to <0.1% (black bars in Fig. 5A), while K. aerogenes recovery was ≥80% for all input ratios tested (red bars in Fig. 5A). K. aerogenes-819-56 inhibited S. aureus recovery dose-dependently, as did donor and commercial strains of *C. koseri* and *M. lincolnii* (black bars in Fig. 5B to E). Collectively, these data suggest that S. aureus survival in nasal medium is thwarted by relative scant amounts of select *Gammaproteobacteria*, including K. aerogenes, *C. koseri*, and *M. lincolnii.*

### Inhibition of S. aureus growth on polarized nasal epithelia by noncarrier-associated *Gammaproteobacteria*.

**FIG 6:**
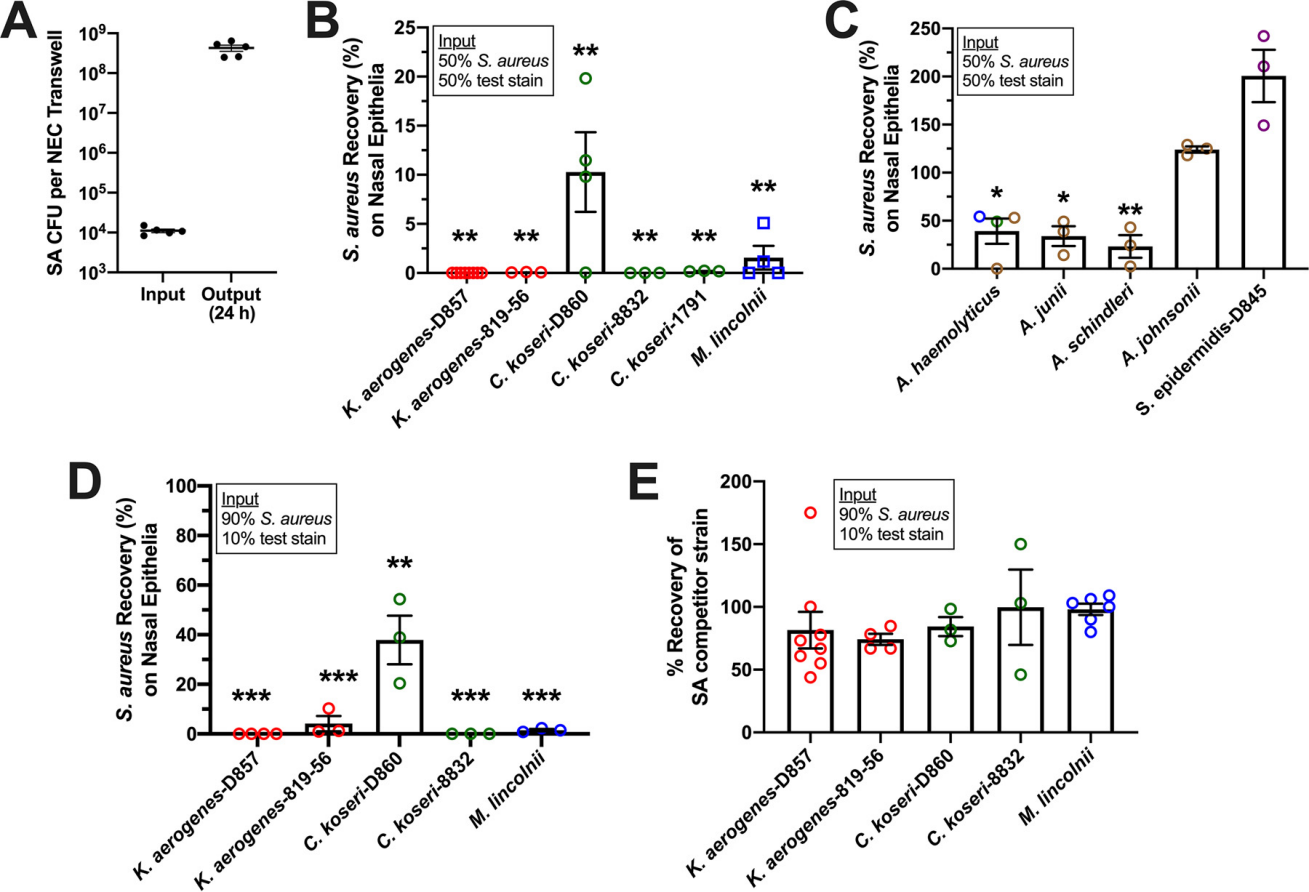
Select *Gammaproteobacteria* inhibit S. aureus survival on nasal cells. S. aureus competition assays were performed as described in Fig. 4 and 5 except that incubations occurred in the apical chamber of Transwells containing polarized primary human nasal epithelial cells. (A) Growth of 5 unique strains of S. aureus on polarized primary nasal epithelia, presented as input and output (24 h) CFU/well. (B and C) S. aureus recovery (%) for incubations of S. aureus mixed with an equivalent input of the indicated strain. *, *P* < 0.05; **, *P* < 0.01 versus S. aureus alone (100% recovery). (D) S. aureus recovery (%) for incubations containing 90% S. aureus + 10% indicated strain. **, *P* < 0.001; ***, *P < *0.0001 versus S. aureus incubated alone (100% recovery). (E) Percent recovery of competitor strains from the 90% S. aureus/10% test strain mixtures presented in panel D. For all panels, bars and error bars represent the mean ± SEM.

To further evaluate promising competitor strains in an environment in which S. aureus thrives, bacterial mixtures in SNM were placed on primary human nasal epithelial cell (NEC) layers that were polarized on porous Transwell inserts. Without antibiotics in the cells or underlying maintenance medium, which contained growth factors and 5% serum, a 10^4^ CFU/Transwell S. aureus inoculum proliferated over 10,000-fold in 24 h (Fig. 6A). When 0.5 × 10^4^ S. aureus CFU/Transwell was incubated with an equivalent amount of test strain, surviving bacteria were determined after 24 h by dilution-plating the apical fluid to selective agars that differentiate S. aureus from the *Gammaproteobacteria* of interest. Both K. aerogenes strains, *C. koser*i-8832 and *C. Koseri*-1791, retained their potent anti-S. aureus activity, reducing recovery to <0.1% (Fig. 6B). *C. koseri*-D860 and *M. lincolnii* reduced S. aureus recovery to 10% and 2%, respectively. Acinetobacter haemolyticus (3 strains), A. junii, and *A. schindleri* permitted 30 to 40% S. aureus recovery (Fig. 6C). Acinetobacter johnsonii and S. epidermidis did not limit S. aureus recovery (Fig. 6C), although they exhibited over 1,000-fold growth on NEC when incubated without S. aureus (not shown). C. pseudodiphtheriticum did not grow on NEC and appeared not to impact S. aureus survival when coincubated (not shown). The five highly effective strains were retested using an input of 9,000 S. aureus CFU + 1,000 competitor CFU and inhibited S. aureus recovery to <1% (K. aerogenes-D857, *C. koseri*-8832), <10% (K. aerogenes-816-56, *M. lincolnii*), and <50% (*C. koseri*-D860, Fig. 6D). While S. aureus recovery was markedly inhibited, detection of the 5 competitor strains was 70 to 100% compared to when they were cultured on Transwells without S. aureus present (Fig. 6E). It should be noted that most S. aureus strains damage epithelia when incubated for 24 h without competing bacterial species. After 24 h of incubation in the presented experiments, visual inspection of the Transwells prior to collection of bacteria (which disrupted the cell layers) showed that cells incubated with S. aureus alone were the most heavily damaged. S. aureus recovery from the basolateral chamber occurred only in wells in which S. aureus was inoculated alone. These experiments suggested that K. aerogenes, *C. koseri*, and *M. lincolnii* limit S. aureus survival in the nasal mucosa.

### K. aerogenes, *C. koseri*, and *M. lincolnii* inhibit S. aureus survival in nasal secretions.

**FIG 7:**
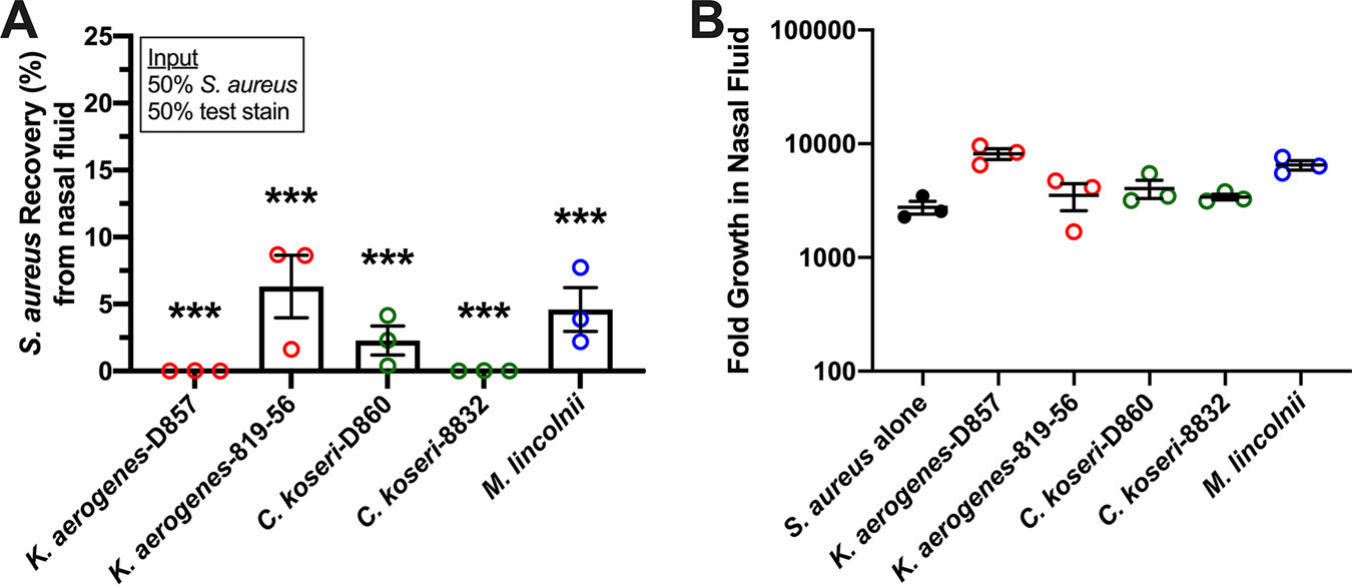
K. aerogenes, *C. koseri*, and *M. lincolnii* inhibit S. aureus survival in nasal secretions. Three unique strains of S. aureus (2 nasal isolates and USA300) were incubated alone or with an equivalent input of the indicated strain in undiluted human nasal fluid which was processed briefly to prevent growth of endogenous microbes. (A) S. aureus recovery (%) for the presented incubations. Bars represent the mean ± SEM; *n* = 3; ***, *P* < 0.0001 versus S. aureus alone (100% recovery). (B) Fold growth in nasal fluid for S. aureus alone (3 strains, black circles) or the indicated competitor (open circles) when mixed with S. aureus. Error bars represent the mean ± SEM.

To test for activity of K. aerogenes, *C. koseri*, and *M. lincolnii* against nasal S. aureus strains in an environment more physiological than SNM, assays were performed similarly to above except that a medium background of human nasal fluid (NF) was used. Whole nasal fluid (no dilution during collection) was processed briefly to eliminate endogenous bacterial CFU, and an NF pool was prepared by mixing equal volumes of processed NF from 10 healthy donors. Treatment wells (0.1 ml NF) were incubated alone (to confirm the absence of CFU after processing), with 10^4^ S. aureus CFU, or with 5,000 S. aureus CFU mixed with 5,000 CFU of test strain. For three different strains of S. aureus (2 nasal isolates and USA300), growth in NF exceeded 1,000-fold in 24 h (Fig. 7). K. aerogenes-D857 and *C. koseri*-8832 reduced S. aureus recovery to <1%, and K. aerogenes-816-56, *M. lincolnii*, and *C. koseri*-D860 reduced S. aureus recovery to <10% (Fig. 7A). Notably, these strains showed the capacity to survive and proliferate in NF while attenuating S. aureus (Fig. 7B). Collectively, the presented experiments have identified resident nasal species that limit S. aureus survival and detection by routine nasal sampling methods. K. aerogenes, *C. koseri*, and *M. lincolnii* were next tested for secreted factors that inhibit S. aureus growth.

### Conditioned medium from NEC plus K. aerogenes and NEC plus *M. lincolnii* cocultures exhibit anti-S. aureus activity mediated by heat-stable <30-kDa proteins.

**FIG 8:**
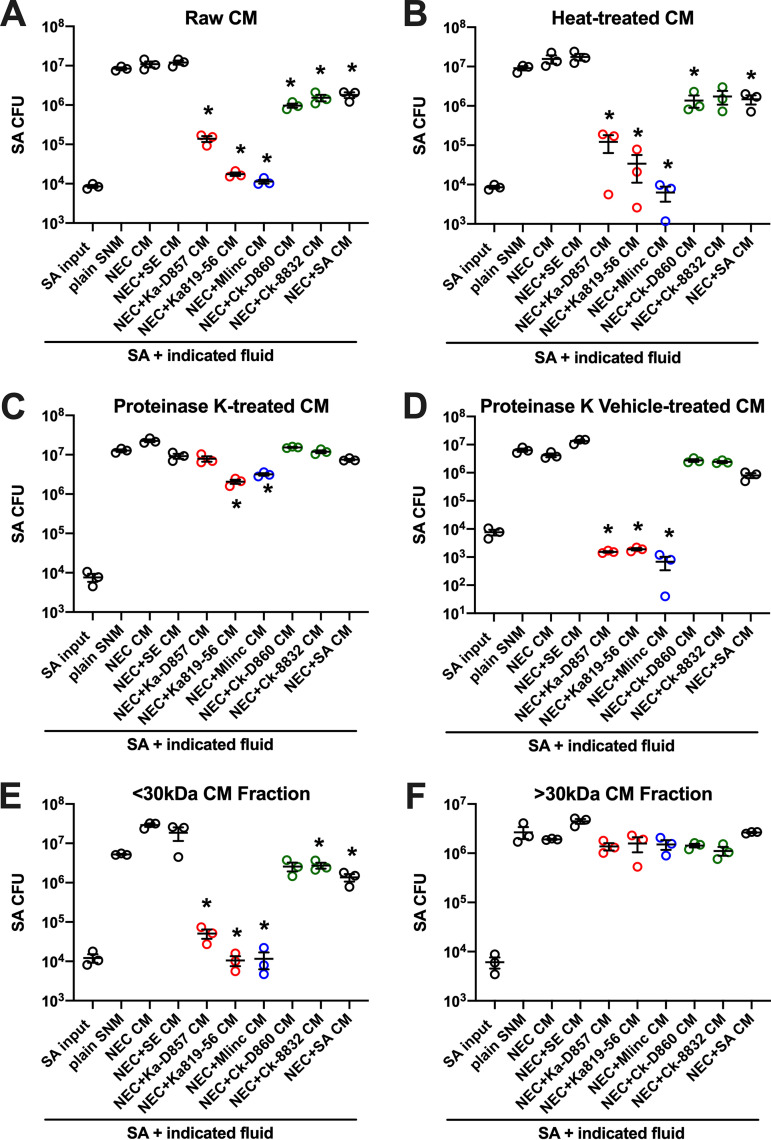
Inhibited growth of S. aureus by heat-stable <30-kDa proteins found in CM from K. aerogenes- and *M. lincolnii*-treated NEC. S. aureus (∼10^4^ CFU) was incubated 24 h in synthetic nasal medium (SNM) or conditioned medium (CM) from primary nasal epithelial cells (NEC) incubated alone or with the indicated bacteria in SNM. SE, S. epidermidis; Ka, K. aerogenes; Ck, *C. koseri*; M. linc, *M. lincolnii*; SA, S. aureus. Collected CM was centrifuged and filter-sterilized and confirmed to be devoid of bacterial CFU prior to experimentation. (A to F) Growth of S. aureus is displayed as CFU determined by agar plating for raw CM (A), 95°C/5 min-treated CM (B), proteinase K-treated CM (C), vehicle (storage solution for proteinase K)-treated CM (D), <30-kDa protein fraction of CM (E), and >30-kDa protein fraction of CM (F). Error bars indicate the mean ± SEM for each treatment group. Each circle represents a unique strain of S. aureus. *, *P < *0.05 versus S. aureus recovery from SNM.

Conditioned medium (CM) was collected from confluent primary NECs incubated alone or with individual bacterial strains for 24 h in SNM. Prior to experiments with S. aureus, CMs were clarified by centrifugation, followed by 0.2 μm filtration, confirmed free of bacterial CFU by agar plating, and frozen in single-use aliquots. The growth of three different strains of S. aureus (nasal 547-14 sequence type 5 [ST5], nasal 512 [ST30], USA300 [ST8]) was evaluated in freshly thawed CM or fresh SNM (∼10^4^ CFU/0.1 ml) after incubation for 24 h. Initial S. aureus inocula (e.g., CFU/well at time 0 h) were confirmed by agar plating and subsequent CFU enumeration. S. aureus survival at 24 h was determined by plating multiple dilutions of each fluid and calculating the output CFU per treatment well (Fig. 8). CM from NEC coincubations with K. aerogenes-D857, K. aerogenes-816-56, and *M. lincolnii* inhibited S. aureus detection by 2 to 3 logs compared to S. aureus recovered from plain SNM, CM from untreated NEC, or CM from NEC plus S. epidermidis coincubation (Fig. 8A). CM from *C. koseri*- and S. aureus-NEC CMs inhibited S. aureus recovery by about 10-fold. This trend was conserved when CMs were heat-treated (95°C/5 min) prior to incubation with S. aureus (Fig. 8B), suggesting the presence of heat-stable anti-S. aureus proteins or peptides. Proteinase K treatment abrogated the effect of K. aerogenes- and *M. lincolnii*-NEC CMs (Fig. 8C), while vehicle treatment of CMs with proteinase K storage solution preserved the anti-S. aureus activity (Fig. 8D). Last, we tested whether <30 kDa proteins mediate the observed anti-S. aureus activity by processing CMs through 30 kDa molecular weight cutoff filters. Figure 8E shows that the anti-S. aureus potency of K. aerogenes- and *M. lincolnii*-NEC CMs is retained in the <30-kDa fraction. S. aureus growth and viability in the >30-kDa CM fractions were similar for all bacterium-NEC combinations (Fig. 8F). CM from *Klebsiella* and *Moraxella* cultures, incubated in SNM but without NEC, markedly inhibited S. aureus recovery compared to uninoculated SNM or CM from NEC cultures (not shown). These data suggest that secreted <30-kDa proteins from nasal K. aerogenes and *M. lincolnii* limit the detection of nasal S. aureus.

## DISCUSSION

The nasal cavity filters thousands of liters of air per day, and the nasal mucosa secretes about a liter of fluid per day (37). Passage of inhaled particles or microorganisms up and out of the nasal cavity or lungs is achieved when mucous-trapped inhalants are moved by epithelial cilia toward the throat, where they are swallowed or expelled by coughing. The anterior nares (nostrils) represent a transition zone from skin to the airway mucosa and are not part of this mucociliary escalator. Thus, the resident microflora of this anatomical region, combined with microbicidal substances, help shape the nasal mucosal immune ecosystem of each host. SANC presents an obvious risk factor for autologous infection, as S. aureus is one of the most common pathogens isolated from skin and soft tissue wounds and surgical sites, and nasally carried strains are usually genetically indistinguishable from strains isolated from infection sites (7, 38, 39). Intermittent and persistent carriers acquire and lose S. aureus strains at similar frequencies (5, 40), but persistent carriers possess a higher nasal S. aureus load than intermittent carriers. This brings into question whether intermittent and noncarriers truly exist or just carry S. aureus below culture detection limits (23, 41). To date, it is not understood why some healthy humans carry detectable levels of nasal S. aureus persistently, intermittently, or seemingly not at all. Metabolomic analysis of nasal secretions showed low levels of potential nutrients and no appreciable differences between SANC and noncarrier secretions (26), and few studies have explored specifically the nasal microbiomes of established carriers versus noncarriers. The positive correlation between nasal S. aureus load and *ex vivo* growth of S. aureus strains in matched nasal fluids from SANC subjects (Fig. 1) suggested that S. aureus CFU detection might relate to the composition and quantity of microbiome-derived soluble proteins contained in nasal fluids. We therefore set out to determine species-level differences in the microbial composition of established SANC and non-SANC nostrils and to test noncarriage-associated microbial community members for activity against S. aureus in an environment mimicking the nasal milieu.

Our multipronged approach included 16S amplicon sequencing of 65 swab samples collected from established noncarrier, intermittent, and persistent SANC nostrils; shotgun metagenomic sequencing of DNA collected freshly from 7 established noncarrier swabs and 6 persistent SANC swabs; and culture-based isolation and identification of nasal species from non-SANC noses. Shotgun DNA sequencing afforded species- and strain-level resolution, and all presumed non-SANC subjects (based on culture results) had detectable S. aureus DNA, while one noncarrier exhibited 11% S. aureus DNA (relative abundance; Fig. 3A). Culture S. aureus-positive noses carried S. aureus ranging in relative abundance between 0.7% and 33%, demonstrating that some people do not harbor high levels of S. aureus DNA but still swab positive persistently, while some presumed “noncarriers” host abundant S. aureus DNA without exhibiting CFU. Others have reported nasal S. aureus DNA to be nearly always present (42, 43), and all people are thought to possess anti-S. aureus antibodies (44). Taken together, these findings support the concept that people are consistently exposed to S. aureus in everyday life and that SANC likely represents a microbial shift condition that can be modulated with probiotics or improved topical anti-S. aureus biologicals once the key influential species are identified.

The *Gammaproteobacteria* genera *Citrobacter* and *Moraxella* were detected using 16S amplicon sequencing and associated with some non-SANC nostrils (Fig. 2A), and both *C. koseri* and *M. lincolnii* were found in greater relative abundance in noncarrier nostrils when assessed by shotgun sequencing (Table 1). K. aerogenes was not as abundant in nostril swabs as *M. lincolnii* or *C. koseri*, but it was associated more with noncarrier noses (Table 1). Notably, we were able to isolate *C. koseri* and K. aerogenes from noncarrier but not carrier noses. These strains as well as purchased nasal strains of *C. koseri*, K. aerogenes, and *M. lincolnii* proliferated upward of 1,000-fold in SNM, indicating that they can adapt to the nutrient-limited environment of the anterior nares (Fig. 4). Collectively, the finding that these species strongly inhibited S. aureus recovery even when the S. aureus:competitor input ratio was 90:10 and the test environment (nasal epithelia or nasal fluid) supported rampant S. aureus growth when no competitor was added (Fig. 5 and 7) suggests that even relative scant amounts of these species might prevent detection of nasal S. aureus by standard swabbing and culture-based procedures. Likewise, the average competitor:S. aureus DNA ratios in established noncarriers were 1:10.5 for K. aerogenes, 1:4 for *C. koseri*, and 5:1 for *M. lincolnii* (Table 1), thus providing a plausible explanation for why these “noncarrier” subjects were swabbed weekly for months to years without detection of a single S. aureus colony. A study of the nasal microbiomes of chronic rhinitis patients receiving intranasal steroids demonstrated that suppression of *Moraxella* spp. was associated with increased abundance of *Staphylococcus* spp. (45). Vaginal microbial profiling showed an inverse relationship between *Klebsiella* spp. prevalence and S. aureus culture positivity (46). Correspondingly, Liu et al. defined seven major nasal community state types (CSTs) and discovered that nasal microbiomes classified as CST2 (*Enterobacteriaceae* [e.g., *Klebsiella* and *Citrobacter*] prevalent) and CST6 (*Moraxella* spp. prevalent) were not likely to contain S. aureus (28). Since CM derived from K. aerogenes and *M. lincolnii* cultures inhibited S. aureus while CM from *C. koseri* did not (Fig. 8), it is apparent that this class of *Gammaproteobacteria* possesses multiple unique mechanisms for limiting survival of nasal S. aureus that warrant further investigation.

Implementation of K. aerogenes or *C. koseri* as nasal therapeutics for S. aureus decolonization might be challenging, as these species are known opportunistic pathogens. While not as dreaded as *K. pneumonia*, K. aerogenes is associated with urinary tract and bloodstream infections in vulnerable populations and is capable of acquiring antibiotic resistance (47). *C. koseri* is common to the intestinal tract, and though seldom lethal, is known to cause a variety of infections in humans. On the other hand, we isolated K. aerogenes, K. oxytoca, and *C. koseri* from nasal swabs collected from healthy volunteers. We noted that topical administration of K. aerogenes, *M. lincolnii*, and *C. koseri* to polarized primary nasal epithelia resulted in less barrier disruption than administration of S. aureus, but our assays were designed for liberation and enumeration of CFU instead of host cell signaling and toxicity studies. A comprehensive analysis of the effects of these species on nasal tissue (e.g., inflammatory markers, epithelial barrier integrity, chemotactic activity) is needed. In the meantime, identification and characterization of anti-S. aureus protein(s) from these species should include plans for chemical (bacterium-free) synthesis of active peptides as well as safety, stability, and functional testing in nasal tissue and against a variety of resident nasal bacteria. *M. lincolnii* has not been well characterized to date, but it is considered a normal resident of the upper respiratory tract. High abundance of nasal *Moraxella* spp. is associated with healthy young children and low rates of respiratory infections (48), and a longstanding healthy S. aureus noncarrier in our cohort was classified as nasal *M. lincolnii* prevalent (>60% relative bacterial DNA abundance). Thus, *M. lincolnii* represents a promising candidate for preclinical testing as a natural S. aureus competitor on mucosal surfaces and perhaps skin lesions. Future studies should also test whether S. aureus can develop resistance to the *Gammaproteobacteria*-derived peptide(s) and whether there is a fitness and/or virulence cost. Given that few novel anti-S. aureus antibiotics have been brought to market over the past several decades, while S. aureus infections continue to burden health care systems worldwide, a renewed effort to exploit commensal species and their peptide products may prove worthwhile.

Numerous reports have indicated roles for *Corynebacterium* spp. and S. epidermidis in favoring the non-SANC state, and mechanisms of interaction between these commensals and S. aureus have been described (30, 33, 49–51). We found that few obvious distinctions between non-SANC, intermittent, and persistent SANC nostrils were indicated by 16S amplicon sequencing aside from the expected trend that *Staphylococcus* abundance appeared to be inversely proportional to *Corynebacterium* abundance in some individuals who were sampled multiple times (underlined stacked bars in Fig. S1). Interestingly, however, wide ranges of *Staphylococcus* and *Corynebacterium* abundance were revealed in both non-SANC and persistent SANC individuals; a persistent SANC nostril contained <10% *Staphylococcus* and *Corynebacterium* relative abundance, while a noncarrier nostril contained >80% *Staphylococcus* relative abundance (Fig. 2A and Fig. S1). These observations suggest that the competitive relationship between *Corynebacterium* spp. and nasal S. aureus is highly complex and not yet well understood.

Shotgun metagenomic sequencing revealed that *Corynebacterium* spp. (particularly *C. accolens*, *C. segmentosum*, and C. aurimucosum) and S. epidermidis were abundant in all noses (Table 1). A potential limitation of our study is that, due to cost considerations, we performed shotgun sequencing on only 13 swab samples (e.g., single HiSeq lane). However, the species-level data uncovered several potential distinctions between persistent- and non-SANC that are in line with recent reports from other groups or shed light on unanswered questions. Noncarrier noses were associated with an abundance of *C. accolens* and *C. segmentosum* compared to >10 other *Corynebacterium* spp., while SANC nostrils were colonized mainly by *C. aurimucosum* (Table 1). Nasal *C. accolens* was determined to be more prevalent in the absence of S. aureus, although during longitudinal sampling, it was unclear if its presence impacted future acquisition of nasal S. aureus (52). *C. aurimucosum* showed mixed results against 3 strains of S. aureus in agar-based zone of clearance assays, suggesting mainly contact-independent bacteriostatic interaction (31). C. pseudodiphtheriticum interaction with S. aureus may be bactericidal or diminish quorum sensing and fitness (33, 49). Our data revealed that C. pseudodiphtheriticum was present in all noncarrier nostrils but only half of carrier noses, supporting the view that this species reduces the rate of detectable SANC. On the other hand, C. pseudodiphtheriticum did not thrive in SNM or inhibit S. aureus in the nutrient background of SNM (Fig. 4) or SNM placed on the apical surface of polarized NEC. This is consistent with the report that C. pseudodiphtheriticum’s niche is the middle meatus/sphenoethmoidal recess and not the anterior nares (34). Nasal S. epidermidis grew in SNM but failed to limit S. aureus growth (Fig. 4 and 6), supporting Liu et al. (28) and our previous work (15) finding that S. epidermidis coexists with S. aureus in the nose. Indeed, we observed CST3 (S. epidermidis prevalent) and CST5 (*Corynebacterium* spp. prevalent) in both the non-SANC and persistent SANC groups, while CST6 (*Moraxella* spp. prevalent) was observed only in a long-term “never” S. aureus-positive subject. Our shotgun sequencing data also corroborated the findings that *S. lugdunensis* and Dolosigranulum pigrum levels trend higher in non-SANC nostrils (28, 32). *S. lugdunensis* produces a peptide antibiotic, “lugdunin,” that inhibited SANC in cotton rats, and a survey of hospitalized patients found that although nasal carriage of *S. lugdunensis* was >3-fold less common than SANC, only 1 of 17 *S. lugdunensis*-positive noses was S. aureus-positive (32). Future studies aimed at defining the anti-S. aureus properties of *D. pigrum* should be undertaken.

It was surprising that only the ratio of Acinetobacter spp. to S. aureus was significantly different between the S. aureus culture-negative versus S. aureus culture-positive swabs (Fig. 3B), considering that overall abundance of Acinetobacter was considerably lower than that of *Corynebacterium*, non-*aureus Staphylococcus*, *Cutibacterium*, and *Dolosigranulum* (Table 1). Six of seven noncarrier noses exhibited an Acinetobacter:S. aureus ratio of >1, while five of six carrier ratios were <1. Interestingly, the carrier nose in which Acinetobacter outnumbered S. aureus (arrow in Fig. 3B) nearly cleared S. aureus according to a swab sample collected 4 days later. To date, only Acinetobacter baumannii has received considerable attention in airway studies due to its frequent association with pneumonia and its propensity for gaining antibiotic resistance. We failed to cultivate Acinetobacter strains from fresh nostril swabs and, thus, purchased them from ATCC and prepared subcultures and then sequenced the 16S gene to reconfirm their identities. We noted that A. baumannii genetic sequences provide the basis for sequence mapping of less well-characterized Acinetobacter spp. and were always included in BLAST results. A recent screen of wound and nasal isolates determined that select strains identified by 16S sequencing as A. baumannii possess contact-dependent activity against S. aureus (31). Our data also suggest contact-dependent S. aureus inhibition, as *A. haemolyticus*, A. junii, and *A. schindleri* all showed some anti-S. aureus activity when incubated with diverse S. aureus strains on polarized nasal epithelia (Fig. 6C), while CM collected from these species did not prevent S. aureus growth (data not shown). We conclude that Acinetobacter spp. warrant further investigation as S. aureus effectors, as they may sequester a vital S. aureus nutrient or achieve bactericidal activity via direct contact. A recent report of a <15% SANC rate in a long-term care home in which residents were heavily colonized with Acinetobacter (53) provides support for this concept.

In conclusion, we discovered members of the *Gammaproteobacteria* class that reside in S. aureus-negative noses and possess anti-S. aureus properties. K. aerogenes, *C. koseri*, and *M. lincolnii* inhibited S. aureus growth and recovery by over 90% in physiologically relevant assays containing synthetic nasal medium, polarized primary nasal epithelia, and nasal secretions. Select Acinetobacter species, including *haemolyticus*, *junii*, and *schindleri*, were found to be slow growing on nasal epithelia compared to S. aureus but still inhibited S. aureus recovery by over 50%. By comparing the conditioned media from bacterium plus epithelium cocultures, we further revealed that K. aerogenes and *M. lincolnii* secrete <30-kDa protein(s) that inhibit S. aureus, while *C. koseri* and Acinetobacter spp. activity against S. aureus appeared to be contact-dependent. Taken together, the presented data provide an explanation for why some people appear to be noncarriers of S. aureus even though all noses contain S. aureus DNA. Further investigation is needed to define the mechanisms of anti-S. aureus activity, but this study introduces the concept that acquisition and loss of detectable SANC might result from fluctuations in the nasal microbiota. Better understanding of nasal microbial community dynamics and how the nasal microbiome controls S. aureus viability and growth is greatly needed in order to design effective decolonization strategies.

## MATERIALS AND METHODS

### Ethics statement.

A protocol for recruitment of healthy participants and collection of nasal swabs and nasal fluid was approved by the Institutional Review Board of the University of Central Florida. Participants reported no adverse effects.

### Sample collection.

The Staphylococcus aureus strains used in this study were collected from healthy, nasally colonized subjects by our laboratory and genotyped by multilocus sequence typing (MLST) and *spa* typing as described previously (5, 54). Specifically, the strains were 547-14 (ST5, t688), 512 (ST30, t012), 528-11 (ST8, t008), 594-5 (ST188, t037), and 713-4 (ST5, t548). USA300 (ST8, t008, multidrug resistant) was acquired from NARSA (now www.beiresources.org). These represented a cross section of the most common sequence types described in our large longitudinal study of over 100 nasal S. aureus carriers (5). In an effort to isolate and identify prevalent nasal strains from established non-S. aureus carriers (>3 months of weekly S. aureus-negative swabs) or intermittent carriers who swabbed S. aureus-negative 1 to 3 days prior, participants sampled each nostril by rotating a sterile polyester-tipped swab around the anterior vestibule 10 times. Swab tips were vortexed in 0.5 ml TSB (Bacto tryptic soy broth; Becton, Dickinson [BD], Franklin Lakes, NJ) to liberate the microbes. From each 0.5-ml mixture of microbes and broth, 0.1 ml was spread onto each of the following agars: BD CHROMagar S. aureus (Fisher Scientific; catalog no. 14-432-41) for identification of S. aureus, nonselective TSA II/5% sheep blood agar (Fisher Scientific; catalog no. B21261X), MacConkey II (Fisher Scientific; catalog no. B21270X) for selection of mainly Gram-negative species, Leeds agar (Hardy Diagnostics; catalog no. G261), or Hoyle’s tellurite agar (1 liter: 10 g Lab Lemco beef extract, 10 g peptone, 5 g NaCl, 15 g agar, 7% horse blood, 0.35 g potassium tellurite; all components from Fisher). On a few collection days early in the study, plating to nutrient agar, plate count agar, and plain TSA occurred instead of plating to MacConkey II, Leeds, and Hoyle’s agars. These nonselective agars yielded mostly *Staphylococcus* CFU. Participants’ nasal fluids were collected by suction catheter as described previously (15, 21), and stored at −80°C immediately.

### Nasal bacterial strain details and stock preparation.

Colonies plucked from agar plates were cultured in 4 ml TSB overnight (37°C/250 rpm). The next day, 1 ml was supplemented with glycerol to 15% and stored at −80°C, and the rest was centrifuged to collect bacteria prior to DNA extraction (Qiagen catalog no. 12224-250; UltraClean microbial DNA isolation kit). 16S PCR was performed (standard 27F-1493R primers), verified by gel electrophoresis, and then sent for Sanger sequencing (27F, 341F, 1493R primers) at Eton BioSciences (Research Triangle Park, NC). Sequencing files were analyzed using Mega7.0.20 software to align and concatenate the PCR product sequences into the full-length 16S gene for BLAST analysis and species identification. Nasal isolates collected and identified in our laboratory were Staphylococcus epidermidis-D845, Klebsiella aerogenes-D857, Klebsiella oxytoca-D860, and Citrobacter koseri-D860. The following nasal isolates were purchased from American Type Culture Collection (ATCC, Manassas, VA): Klebsiella aerogenes-819-56 (catalog no. 13048), Moraxella lincolnii-LMG5127 (catalog no. 51388), Citrobacter koseri-8832 (catalog no. 25410), Citrobacter koseri-1791 (catalog no. 25408), Acinetobacter haemolyticus (3 strains: catalog no. 19194, 17906, 17907), Acinetobacter johnsonii (catalog no. 17909), Acinetobacter lwoffii (catalog no. 15309), Acinetobacter junii (catalog no. 17908), Acinetobacter schindleri (catalog no. BAA-618), Staphylococcus epidermidis-11047 (catalog no. 14990), and Corynebacterium pseudodiphtheriticum (catalog no. 10700). Vials were cultured according to ATCC instructions, and bacteria from this first overnight culture, as well as several colonies from initial agar plates, were stored as glycerol stocks at −80°C. Since liquid culture (37°C/250 rpm) in rich broth (e.g., TSB, brain heart infusion [BHI]) is not physiologically relevant to the nutrient limitation experienced by nasal microflora, all strains (S. aureus and putative competitors) were propagated for use in experiments via culture (24 h/37°C/5% CO_2_) in a 100-mm dish of confluent primary nasal epithelial cells containing 10 ml of synthetic nasal medium (26), as described below. Nasal cell-free, washed (Hanks’ balanced salt solution [HBSS]/0.1% bovine serum albumin [BSA]) bacteria were flash frozen in liquid nitrogen and stored at −80°C in aliquots (typically, 0.3 to 1 × 10^6^ CFU/μl in HBSS/0.1% BSA) for ease of use on experiment days. At time 0 h of each experiment, a portion of each bacterium stock was plated on CHROMagar S. aureus, MacConkey II, and TSA II/5% blood agars to confirm that (i) all S. aureus strains made mauve colonies on CHROMagar S. aureus and golden colonies on TSA II/5% blood agar, and failed to make any colonies on MacConkey II agar and (ii) competitors could be easily enumerated on MacConkey II (all *Proteobacteria*) or TSA II/5% blood (S. epidermidis and C. pseudodiphtheriticum) but failed to produce CFU on CHROMagar S. aureus.

### Antibiotic resistance testing of nasal bacterial strains.

Donor S. aureus and *Proteobacteria* strains were tested for functional antibiotic resistance by performing turbidity (growth) assays (55). Isolates were diluted to 10^4^ CFU/90 μl in Mueller-Hinton broth (Millipore Sigma, St. Louis, MO) containing 5% sucrose and loaded onto a 96-well (96W) culture plate. Then, 10 μl of 2-fold serial dilutions of either mupirocin, tobramycin, gentamicin, penicillin-streptomycin, or vehicle (volume-matched dimethyl sulfoxide [DMSO] or phosphate-buffered saline [PBS]) were added to the diluted S. aureus so that the final concentration of antibiotic ranged from 1 to 100 μg/ml. Each culture plate was covered with ThermoSeal A film (Excel Scientific, Inc., catalog no. TSA-100) and placed into a SpectraMax 190 microplate reader (Molecular Devices, Sunnyvale, CA) programmed for a 16-h kinetic assay at 37°C. Turbidity measurements (optical density [OD] at 550 nm) were taken every 5 min following 15 sec of agitation. Growth curves (OD at 550 nm plotted against time) were generated for all wells, and both the input and 16-h incubations were plated on TSA II/5% sheep blood agar for enumeration. All strains grew to 10^7^ to 10^8^ CFU/0.1 ml in antibiotic-free medium. All S. aureus strains were killed in the presence of 1 μg/ml mupirocin, while all nasal *Proteobacteria* tested were mupirocin resistant. All nasal strains tested were killed in the presence of 1 to 10 μg/ml tobramycin, ≥10 μg/ml gentamicin, and penicillin (100 IU/ml)-streptomycin (100 μg/ml).

### Next-generation sequencing (NGS) of nasal swabs.

Nasal swab samples were first assayed for taxonomic composition by sequencing the V3-V4 variable region of the 16S rRNA gene (primers 341F and 785R [56]) on Illumina’s MiSeq platform. In brief, DNA was extracted from nasal swabs using the ZymoBIOMICS DNA microprep kit (Zymo Research Corp., Irvine, CA). PCR was carried out according to Illumina’s instructions (16S metagenomic sequencing library preparation protocol) using an amplicon primer with specified overhangs, and bead cleanup was followed by indexing with uniquely barcoded Illumina adapter sequences (Nextera XT index primers). After a second bead purification of indexed amplicons, samples were quantified (Quant-iT PicoGreen; Molecular Probes, Inc., Eugene, OR) and pooled according to equimolar content. The final sequencing pool was checked for quality using TapeStation 4200 (Agilent) and sent to the University of Florida’s Interdisciplinary Center for Biotechnology Research (ICBR) for MiSeq sequencing (2 × 300 bp). The sequence data were processed using the USEARCH/UPARSE program suite (57, 58) to identify operational taxonomic units (OTUs), and OTU taxonomic assignments were made using a naive Bayesian classifier implemented in mothur (59, 60). OTUs with the same genus assignment were combined to identify the relative abundances of bacterial genera in the nasal microbiomes of a cohort of healthy human volunteers determined to be either long-term (“persistent”), intermittent, or noncarriers of nasal S. aureus (*n* = 65). The OTU data were used to calculate alpha diversity (Shannon diversity index). The genus-level data were used to cluster the samples based on the DMM framework (35). Since species- and strain-level differences cannot be inferred by studying 16S rRNA genes alone, we performed shotgun metagenomics analysis of swab samples from carriers and noncarriers to garner species/strain-level details. Fresh nasal swabs were collected from volunteers whose SANC status had been established for at least 3 months and was confirmed by culture 1 to 3 days prior, and microbial DNA was enriched by depleting cell-free and mammalian DNA prior to microbial DNA extraction (MolYsis Basic5 microbial DNA enrichment kit; Oasis Diagnostics Corp.). Nextera XT libraries (Illumina, Inc., San Diego, CA) were prepared and indexed according to company instructions, sizes and quantity verified using an Agilent 4200 TapeStation system and high sensitivity (HS) D1000 reagents, and pooled. Altogether, 14 samples (7 noncarriers/6 carriers/1 microbial community standard [Zymo Research Corp., Irvine, CA]) were sequenced (HiSeq 2 × 150-bp/dual index/1 lane; Genewiz, Inc.), yielding 462 million reads with a mean quality score of 36.72. After trimming of adapter sequences and low-quality bases, the reads were mapped to the human genome. Unmapped reads from this step were subsequently mapped against a collection of >10,000 microbial genomes (NCBI RefSeq). This read mapping information was used to estimate relative abundances of genomes (strains) in each sample using an expectation maximization algorithm (61, 62). The most abundant genomes (≥0.01 genome abundance) accounted for >150 species, ∼90 of which were common to most nasal swabs. For comparisons between noses in which certain species were not detected, the relative abundance was set at 0.005.

### Preparation of simulated nasal medium and first-pass S. aureus competitor screening.

Synthetic nasal medium (SNM) was prepared as described for “SNM3” by Krismer et al. (26). All amino acids, organic acids, trace elements, cofactors, and 2,2′-bipyridine were purchased from Millipore Sigma (St. Louis, MO). Sodium phosphate, potassium phosphate, sodium chloride, potassium chloride, and magnesium sulfate were purchased from Fisher Scientific (part of Thermo Fisher, Waltham, MA). Stock solutions of the components were prepared at 40 to 2,000-fold and stored at 4°C in the dark for ease of use and standardization of individual batches. SNM was demonstrated to mimic the environment of the nasal mucosa and support S. aureus growth and gene expression observed in human nasal S. aureus colonization (26). For all presented experiments, we utilized SNM supplemented with 0.1% (vol/vol) human serum (GeminiBio, West Sacramento, CA). In testing the growth capacities of various nasal microbial community inhabitants in SNM/0.1% serum, inputs ranging from 10,000 to 100,000 CFU per 0.5 ml were incubated 24 h in 24W tissue culture dishes at 37°C/5% CO_2_. All *Staphylococcus* strains and most *Klebsiella*, *Citrobacter*, and *Moraxella* strains proliferated >100-fold at these initial densities, and the first round of competition assays tested 50,000 CFU S. aureus versus 50,000 CFU competitor in 0.5 ml SNM/0.1% serum. When different ratios were used, the total bacterial input was held constant at 100,000 CFU; 90:10 was defined as 90,000 CFU S. aureus versus 10,000 CFU competitor, 70:30 meant 70,000 CFU S. aureus versus 30,000 CFU competitor, etc. In every experiment, a portion of the prepared bacterial solutions was plated on CHROMagar S. aureus, MacConkey II, and TSA II/5% blood to confirm the input concentration and colony phenotypes. At 24 h, treatment wells were mixed by pipetting up and down and were plated in serial dilutions to the same agars as follows: CHROMagar S. aureus to enumerate S. aureus CFU, MacConkey II to enumerate *Klebsiella*, *Citrobacter*, *Moraxella*, and select Acinetobacter CFU, and TSA II/5% blood agar for visualization of all strains’ CFU.

### Air-liquid interface primary nasal epithelial cell culture.

Human primary nasal epithelial cells (NEC; catalog no. T4014) and Prigrow I medium (catalog no. TM001) were purchased from Applied Biological Materials, Inc. (Richmond, BC, Canada). Cells were maintained and expanded on collagen-coated tissue culture plates using a medium of Prigrow I supplemented with 5% fetal bovine serum (FBS) (Peak Serum, Wellington, CO). A 30× collagen solution was purchased from Advanced BioMatrix (San Diego, CA) and diluted to 1× with sterile, tissue-culture-grade water prior to each use. Each 100-mm dish was coated with 5 ml of 1× collagen for at least 2 h (37°C/5% CO_2_); then, liquid was removed and plates were rinsed with 5 ml PBS before cells were seeded. Early passage NEC were maintained in the presence of penicillin and streptomycin (purchased as 100× mix from Gibco/Thermo Fisher), and aliquots of cells were frozen and stored in liquid nitrogen for future use at passages 3 to 7. The freeze medium contained 50% FBS/40% Prigrow I/10% DMSO. To prepare for coculture of NEC with bacteria at the air-liquid interface (ALI), cells from a confluent 100-mm dish were seeded at 0.5 ml per well to a 12-well PET Transwell dish (Corning; catalog no. 3460). These 0.4-μm pore Transwells were precoated with collagen as described above. The day after cell seeding, an ALI medium was prepared by supplementing antibiotic-free maintenance medium with 0.4 mM calcium chloride and used for daily medium changes (1 ml per underlay, 0.3 ml per overlay until day 3 or 4, when medium was removed from the apical cell surface). Treatments were performed within 4 to 7 days following ALI exposure (typically day 8 to 11 after seeding), when each well exhibited a nasal cell layer capable of sealing the basal medium away from the apical compartment of the Transwell and transepithelial electrical resistance (TEER) exceeded 280 Ω·cm^2^. TEER was measured using the EVOM^2^ voltohmmeter and EndOhm-12 chamber for 12-mm culture cups (World Precision Instruments, Sarasota, FL).

### Coculture of S. aureus and competitors on NEC Transwells.

Immediately prior to treatment, Transwell underlay medium was removed from each well and replenished with fresh medium (Prigrow I with 5% FBS and 0.4 mM CaCl_2_), and apical surfaces were confirmed to be moist but devoid of underlay medium that can seep through disrupted epithelia. Leaky Transwells were not used for experiments because excessive seepage of underlay medium into the apical chamber would promote bacterial overgrowth. Each bacterial stock (preparation described above) was thawed rapidly by swirling in a 37°C water bath and then diluted with SNM such that each Transwell was treated with a total of 10,000 CFU in 100 μl. Accordingly, a ratio of 50:50 (shown in Fig. 6B and C) means that 5,000 CFU of S. aureus was mixed with 5,000 CFU of test strain, a ratio of 90:10 (shown in Fig. 6D) means that 9,000 CFU of S. aureus was mixed with 1,000 CFU of test strain. Serial dilutions of the prepared bacteria were agar plated to record the actual CFU/well at time zero. After 24 h (37°C/5% CO_2_), apical fluids (including disrupted/floating cells) were transferred to microtubes, and the remaining cells were vigorously washed and transferred to the same collection tube using ice-cold Hanks’ balanced salt solution (HBSS). Underlays were collected in their own microtubes and stored on ice until agar plating for bacterial enumeration. Apical fluid-rinse mixtures were centrifuged at 300 × *g* (4°C, 5 min) to separate epithelial cellular debris from bacteria. Bacteria were then collected by centrifugation at 10,000 × *g* (4°C, 5 min), suspended in HBSS, and dilution-plated to various agar formulations—CHROMagar S. aureus, TSA II/5% sheep blood, and MacConkey II.

### Nasal fluid processing and incubation with S. aureus and competitor strains.

Since collected nasal fluids (NF) were immediately frozen as described above, processing occurred at the time of first thaw. Fluids were pulse-sonicated (1 sec × 30) on wet ice using power level 2 (Fisher Scientific sonic dismembrator model 100), clarified by centrifugation (13,000 rpm/5 min/4°C), and then incubated at 55°C for 20 min to prevent the growth of endogenous bacteria. This method was determined to be preferrable to filtration, as 0.2 μm filtration of NF was determined to result in 20% loss of total protein. Compared to heat treatment at 95°C for 5 min, the 55°C/20 min method was also found to preserve protein composition, as evidenced from gel analysis. NF was confirmed free of live bacteria by direct plating to TSA II/5% blood agar and observing no visible CFU after 48 h (37°C bacterial oven). To test for activity of *C. koseri*, K. aerogenes, and *M. lincolnii* against nasal S. aureus strains in NF, processed NF was pooled from 10 donors in order to create a volume that would enable sufficient experimental replicates. Each bacterial strain was diluted in pooled NF to achieve an incubation volume of 100 μl containing 5,000 CFU of each bacterium pair (e.g., *C. koseri* versus S. aureus) or 10,000 CFU of S. aureus alone. Mixtures were incubated at 37°C/5% CO_2_ in 96W tissue culture plates for 24 h before dilution plating to CHROMagar S. aureus or MacConkey II to enumerate S. aureus versus *Citrobacter*, *Klebsiella*, or *Moraxella* colonies.

### Preparation and processing of conditioned medium for evaluation of anti-S. aureus effectors.

To prepare conditioned medium (CM) from bacteria plus NEC cocultures, 100-mm tissue culture dishes containing a confluent monolayer of primary NEC were treated with 8 ml/dish of SNM/0.1% human serum containing ∼4 × 10^6^ CFU (multiplicity of infection [MOI] ∼1) of the bacterium of interest (e.g., *C. koseri*, K. aerogenes, *M. lincolnii*, etc.). Immediately after the treated dishes were placed at 37°C/5% CO_2_, serial dilutions of bacteria were plated on agar to confirm the inoculum. After 24 h, each dish was scraped with a cell lifter, and the combined mixture of cells, bacteria, and supernatant was transferred to conical tubes. After centrifugation at 300 × *g* (4°C, 5 min), which pelleted NEC, the resulting supernatant was centrifuged again at 10,000 × *g* (4°C, 5 min) to collect bacteria. This clarified supernatant was then passed through a 0.2-μm polyethersulfone (PES) syringe filter (Corning; catalog no. 431229) to remove residual bacteria, and the resulting CM was tested for bacterial growth by direct plating to TSA II/0.5% blood agar plates and stored in aliquots at −80°C until use. CM containing bacteria alone was made in exactly the same way, using 100-mm tissue culture dishes but no epithelial cells. Uninoculated NEC (no bacteria) CM was made in parallel with NEC plus bacteria coincubations. Anti-S. aureus activity of individual CM preparations was evaluated by adding 10 μl (10,000 CFU) of freshly thawed and diluted S. aureus to 90 μl of freshly thawed CM in a 96W tissue culture dish and incubating it at 37°C/5% CO_2_ for 24 h. Bacteria were enumerated by plating serial dilutions on agar plates, and fold growth was calculated (T_24h_/T_0h(input)_).

To characterize the active components of CM, aliquots were (i) heat-treated (95°C/5 min) and compared with freshly thawed CM that was not subjected to heat, (ii) treated with 8 μg (∼10% of total protein) proteinase K (Zymo Research, Irvine, CA) versus vehicle (proteinase K storage solution), or (iii) centrifuged through 30-kDa molecular weight cutoff filters according to the manufacturer’s instructions (Microcon YM30; Millipore Corp.). Cutoff at 30 kDa was chosen because previous characterization of epithelia plus bacteria CM showed that some <20-kDa cytokines were found in the retentate instead of the expected flowthrough (63). This suggested that in nondenaturing conditions, some proteins aggregate prior to molecular weight cutoff. Filters were washed with sterile water, and the flowthroughs were pooled and concentrated to the original volume using vacuum centrifugation (SPD 1010 SpeedVac; Thermo Fisher). Following the filter washes, the desalted retentates (>30-kDa fraction) were collected and restored to the original volume with SNM/0.1% serum. These processed CMs were inoculated with S. aureus as described above, and S. aureus CFU were quantified after incubation at 37°C/5% CO_2_ for 24 h.

### Statistical analysis.

Data were analyzed using GraphPad Prism 8 software (GraphPad Software, La Jolla, CA). In most cases, S. aureus counts (CFU/treatment well) were expressed as percent recovery; S. aureus CFU yield for S. aureus alone (no competitor) were set at 100% and compared to S. aureus yield when admixed with test strains. In Fig. 4 and 6 to 8, data were analyzed by one-way analysis of variance (ANOVA). For Fig. 3, groups were compared using unpaired *t* tests (two-tailed). Correlations (Pearson r, R^2^, *P* value in Fig. 1) between nasal S. aureus counts and S. aureus growth *in vitro* were considered significant if *P* < 0.05, and linear regression line was significant for a nonzero slope.
