# Supplementary figures and images for: Identification of Nasal Gammaproteobacteria with Potent Activity against Staphylococcus aureus: Novel Insights into the “Noncarrier” State
Source: mSphere. 2021 Jan 6;6(1):e01015-20. doi: 10.1128/mSphere.01015-20 (PMC7802429; doi:10.1128/mSphere.01015-20)

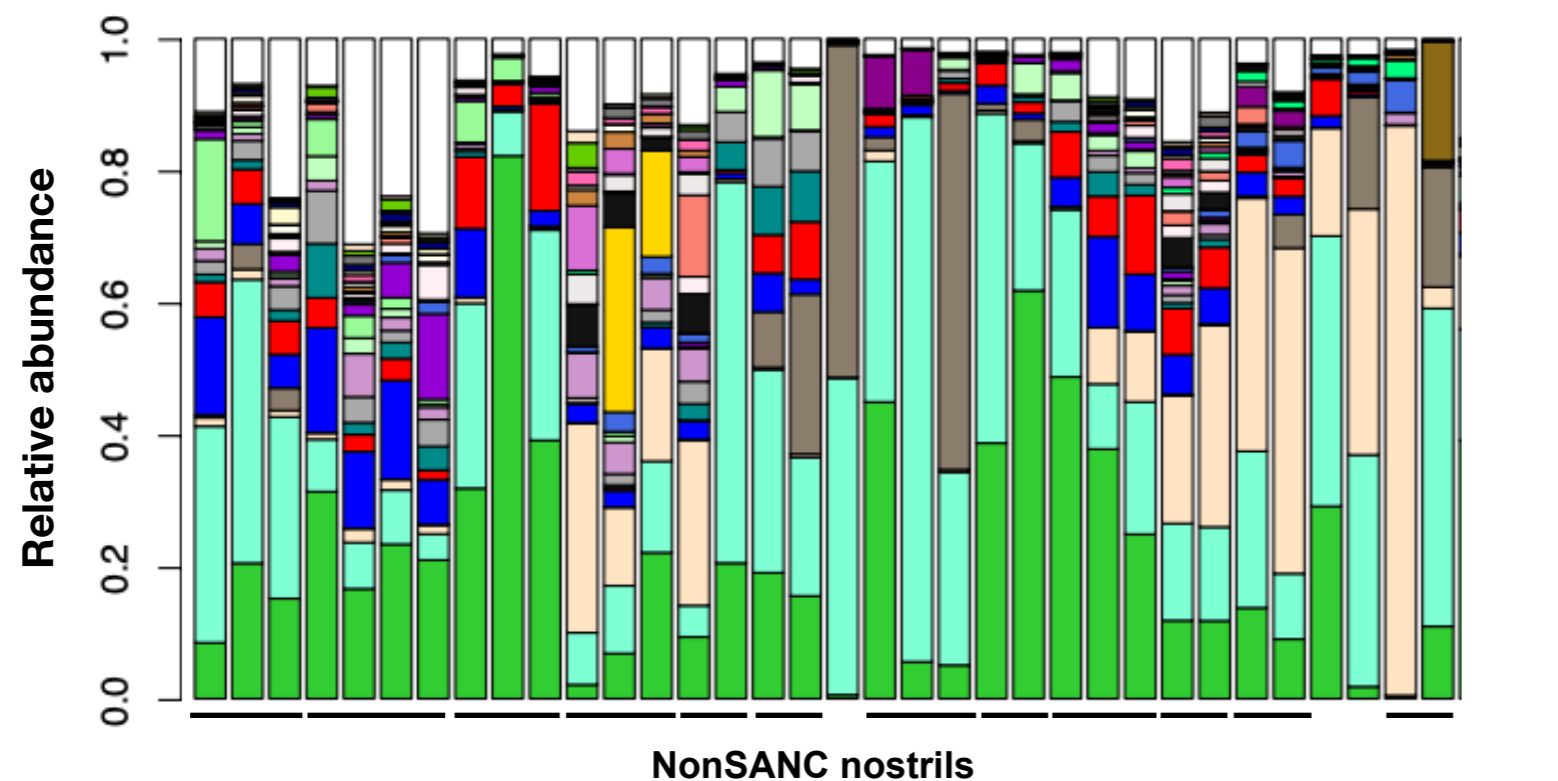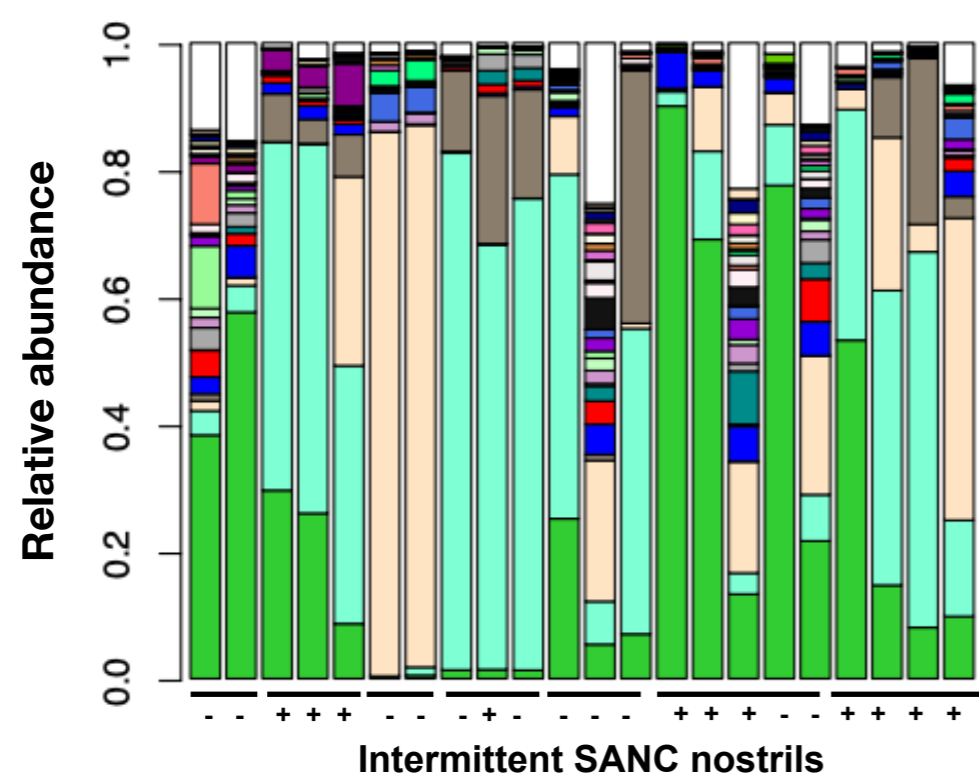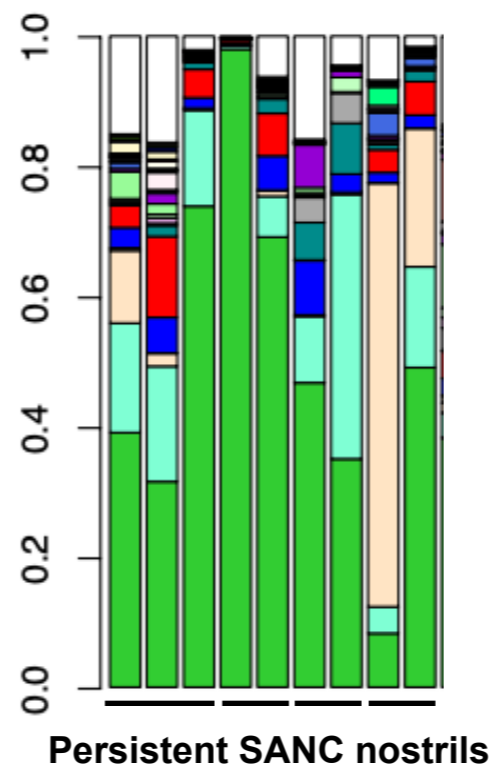

Supplement: FIG S1 [file mSphere.01015-20-sf001.pdf]

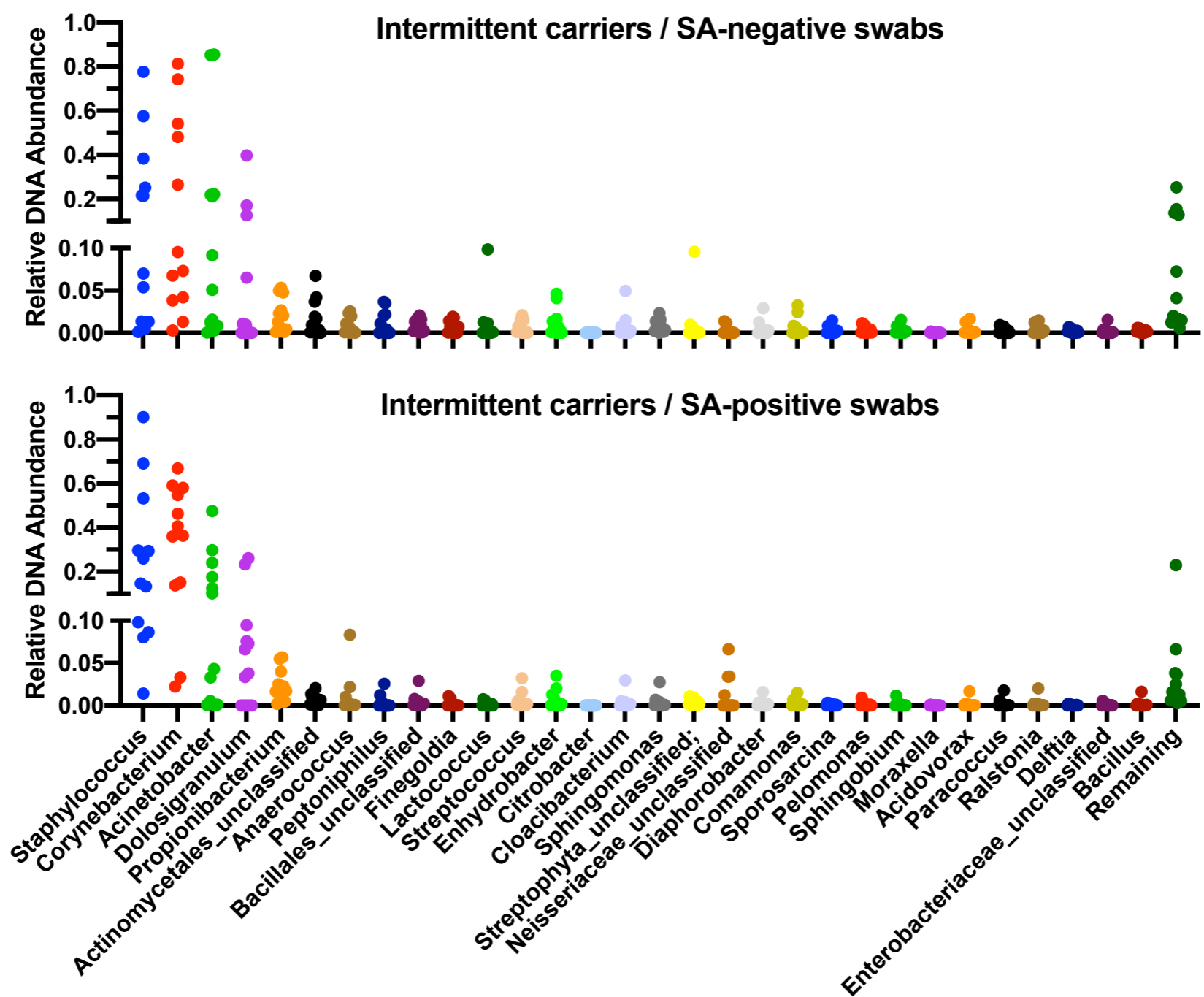

Supplement: FIG S2 [file mSphere.01015-20-sf002.pdf]
